# Phytochemical Profiling and Biological Activities of *Quercus* sp. Galls (Oak Galls): A Systematic Review of Studies Published in the Last 5 Years

**DOI:** 10.3390/plants12223873

**Published:** 2023-11-16

**Authors:** Roxana Banc, Marius Emil Rusu, Lorena Filip, Daniela-Saveta Popa

**Affiliations:** 1 Department of Bromatology, Hygiene, Nutrition, Faculty of Pharmacy, “Iuliu Hațieganu” University of Medicine and Pharmacy, 6 Pasteur Street, 400349 Cluj-Napoca, Romania; roxana.banc@umfcluj.ro (R.B.); lfilip@umfcluj.ro (L.F.); 2 Department of Pharmaceutical Technology and Biopharmaceutics, Faculty of Pharmacy, “Iuliu Hațieganu” University of Medicine and Pharmacy, 12 Ion Creangǎ Street, 400010 Cluj-Napoca, Romania; 3 Department of Toxicology, Faculty of Pharmacy, “Iuliu Hațieganu” University of Medicine and Pharmacy, 6 Pasteur Street, 400349 Cluj-Napoca, Romania; dpopa@umfcluj.ro

**Keywords:** *Quercus*, oak galls, antioxidants, anti-aging, bioactive phytochemicals, phenolic compounds, pharmacological properties, in vitro, in vivo, toxicity

## Abstract

*Quercus* species have been widely used in traditional medicine, and recently, researchers’ attention has focused on galls of the genus *Quercus* as a source of health-promoting phytochemicals. This review presents a summary of the most recent findings on the phytochemistry and bioactivity of oak galls, following the screening of scientific papers published in two relevant databases, PubMed and Embase, between January 2018 and June 2023. The oak galls are rich in active compounds, mostly gallotannins and phenolic acids. Due to these secondary metabolites, the reviewed studies have demonstrated a wide range of biological activities, including antioxidant and anti-inflammatory actions, antimicrobial properties, tissue-protective effects, and antitumor, anti-aging, and hypoglycemic potential. Thus, oak galls are a promising natural matrix, to be considered in obtaining pharmaceutical and cosmetic preparations used in anti-aging strategies and, together with medications, in the management of age-related diseases. In further evaluations, the valuable functional properties of oak galls, reported mostly in preclinical studies, should be confirmed with clinical studies that would also take into account the potential health risks of their use.

## 1. Introduction

Oak is a plant belonging to the genus *Quercus* of the family Fagaceae and it includes over 200 species, which differ in morphology, from tremendous trees to shrubs [1,2]. Regarding the topic of our study, two essential members of this genus are *Q. infectoria* G. Olivier, also known as gall oak [3] or Aleppo oak [4], a small tree or shrub about 2.5 m high [5], growing in countries such as Cyprus, Greece, Turkey, Egypt, Iraq, Iran, Saudi Arabia, Syria, Malaysia, and some parts of India [3,5,6,7], as well as *Q. brantii* Lindl., the most common species in Iran [1].

An important source of polyphenols of *Quercus* sp. is represented by galls (called “oak galls”, “Turkish galls”, “gallnuts”, “nutgalls”, “Mecca galls”, “Aleppo galls”, or “Galla Turcica”), abnormal outgrowths of plant tissue, round in shape, and formed on the leaves, buds, flowers, and young branches, as a result of the sting and laying of eggs by the female gall wasps, *Cynips gallae tinctoriae* and *Adleria gallae-tinctoria* [2,6,7,8].

Historically, *Quercus* sp. galls have been used for millennia in both Western and Eastern cultures as traditional remedies to treat inflammatory conditions, including diarrhea and dysentery, stomach aches, toothaches, and tooth decay, as well as in postpartum care, in combating metabolic abnormalities and oxidative stress-related diseases [1,6,9,10]. In addition to medicinal use, the industrial use of *Quercus* sp. galls dates back to ancient times. Thus, the importance of these species of *Quercus* throughout history is confirmed in medieval manuscripts, galls being used for tanning leather, as dyeing agents for paintings, and natural dyes for carpet yarns, respectively, as a component of ink [1,4,5].

Pharmacologically, *Quercus* sp. galls have been reported to possess strong antibacterial, antioxidant, and anti-inflammatory activities, and also antitumor, antifungal, antiviral, antiprotozoal, antiamoebic, antiulcer, larvicidal, tooth and gum tonic, antipyretic, analgesic/local anesthetic, antidiabetic, cardioprotective, hepatoprotective, antiparkinsonian, antitremor, and accelerated wound healing effects [1,3,4,8,9,11,12]. Despite the multiple therapeutic properties, the long-term intake of gallnuts in high doses is not recommended. Due to the astringent effect of hydrolyzable tannins, they can cause adverse effects, such as irritation of the gastric mucosa, nausea, and vomiting [13]. Also, galls can aggravate lung and throat disorders, such as hoarseness and cough, and can cause anemia and dyspepsia, through the chelation of metal ions, respectively, and the inhibition of digestive enzymes, by tannins [5,13].

In recent years, the search for natural products to prevent or treat diseases is increasing. Among these products, oak galls have attracted the attention of researchers through the biological activities demonstrated both in vitro and in vivo, which are related to the chemical composition rich in antioxidant phenolic compounds [10,12,14,15,16,17]. Compared to other galls, the galls of the genus *Quercus* stand out for the highest level of tannins (50–70%) [1,4]. In addition to tannins, the diverse phenolic profile mainly includes numerous flavonoids and simple phenolic compounds, such as phenolic acids, hydroxyphenols and coumarins, and in a smaller number, representatives from other groups of phenolic compounds, i.e., phenolic aldehydes, naphthodianthrones, acyl-phloroglucinols, phenolic alcohols, and stilbenes [9,10,18,19,20,21].

Through the valuable antioxidant phytochemicals, oak galls could exert their actions and potential effectiveness with fewer side effects and adverse reactions, and lower costs for the population, compared to drugs. Although some articles have been published in this field, to the best of our knowledge, there is no comprehensive and up-to-date analysis of data regarding the *Quercus* sp. galls, with particular focus on the phytochemical profile and biological activities. In this context, the present systematic review integrates in vitro and in vivo studies, published in the last 5 years, and provides an insight into the potential of *Quercus* sp. galls as a source of bioactive secondary metabolites and the relevance of their use in the treatment of various pathologies.

## 2. Methods and Materials 

**Figure 1:**
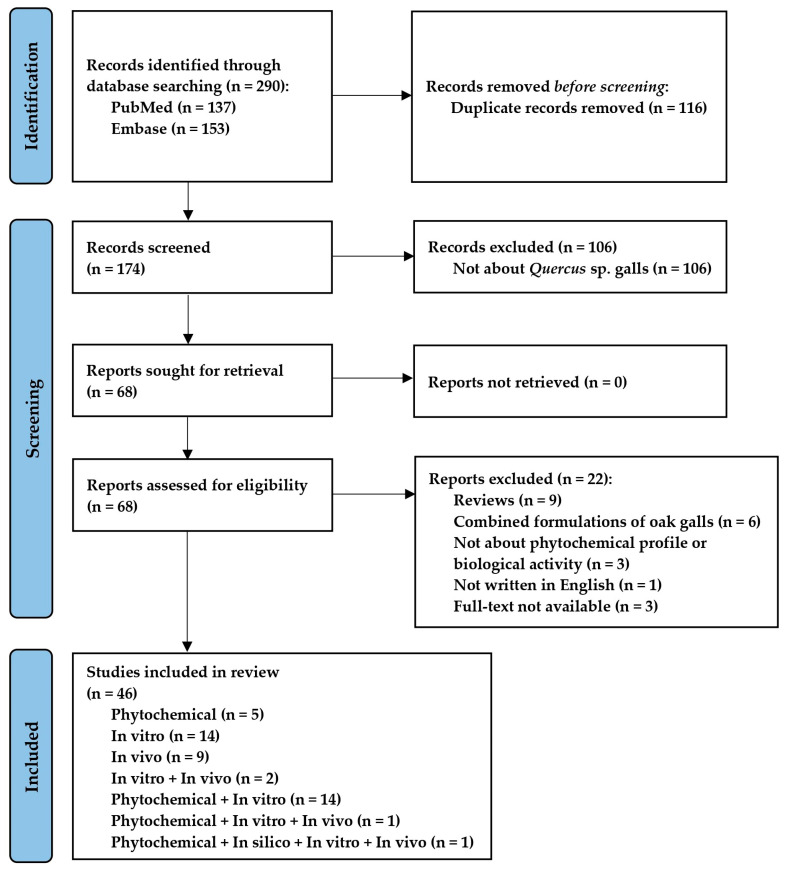
PRISMA flow diagram. Synthesis of the bibliographic analysis.

This systematic review followed the Preferred Reporting Items for Systematic Reviews and Meta-Analyses (PRISMA) guidelines [22] (Figure 1), and the design was registered in INPLASY on 4 October 2023. The registration code is INPLASY2023100012, with DOI 10.37766/inplasy2023.10.0012, https://inplasy.com/inplasy-2023-10-0012/ (accessed on 4 October 2023).

### 2.1. Focus Question

The question to be answered in this systematic review is the following: what are the biological activities shown by the bioactive metabolites of *Quercus* sp. galls in the in vitro and in vivo studies of the last 5 years?

### 2.2. Information Sources

A bibliographic investigation was carried out in PubMed and Embase databases, searching for articles describing the phytochemical profile and biological activity of oak galls published from 1 January 2018 to 30 June 2023. In order to conduct exhaustive research, the bibliographies of the included studies and recent reviews were also examined.

### 2.3. Search Strategy

For the purpose of searching the databases, we made use of a combination of free-text words, as well as their synonyms, singular and plural versions, and thesaurus words (Medical Subject Headings for PubMed: (“quercus” [MeSH Terms] OR “quercus” [All Fields] OR “oak” [All Fields] OR “quercus infectoria” [All Fields] OR “quercus brantii” [All Fields]) AND (“gall” [All Fields] OR “galls” [All Fields] OR “oak gall” [All Fields] OR “gallnuts” [All Fields] OR “nutgall” [All Fields]), and Emtree for Embase: (‘quercus’/exp OR ‘quercus’ OR ‘oak’ OR ‘quercus infectoria’ OR ‘quercus brantii’) AND (‘gall’ OR ‘galls’ OR ‘oak gall’ OR ‘gallnuts’ OR ‘nutgall’)).

### 2.4. Eligibility Criteria 

After the preliminary screening and the elimination of duplicates, the full texts of possibly relevant articles were retrieved and then evaluated to determine whether or not they qualified for inclusion in the review.

The inclusion criteria were (1) experimental studies to identify and/or quantify phytochemical compounds; (2) in vitro and in vivo biological activity studies.

The exclusion criteria were (1) reviews and meta-analyses; (2) secondary studies (i.e., editorials, commentaries, letters to the editor, conference abstracts, or any other publications without original data); (3) studies investigating other types of galls than those collected from *Quercus* sp.; (4) duplicate studies or databases; (5) studies not written in English; and (6) publications with full text not available and the corresponding author could not be contacted.

### 2.5. Selection Process 

Three of the authors performed the literature search, removed duplicate articles, and examined titles and abstracts according to eligibility criteria. Two independent reviewers (R.B. and M.E.R.) conducted the literature search, removed duplicate articles, and carried out the screening of articles according to eligibility criteria. After the titles and abstracts of the extracted references were checked for relevance, the full texts of all potentially eligible articles were screened against the inclusion/exclusion criteria. In case of discrepancies, the disagreements were resolved between them or by a third reviewer (D.-S.P.) who decided whether the study met the inclusion criteria. Furthermore, if essential data for the review were missing, the corresponding author was contacted to obtain the complete information.

### 2.6. Data Collection

Using structured tables, the key data from each study were extracted according to the following descriptive indices: (1) publication characteristics—authors, year of publication, country; (2) study purpose; (3) study type—phytochemical composition study, in vitro study/biological systems analysis, and in vivo study/animal models/with humans; (4) information about the oak gall treatment—plant species used, plant material/extract/formulation type, dose, frequency of administration and treatment in the control group, route of administration; (5) study outcomes. Data from included studies were collected by one reviewer (R.B.) and cross-checked by two others (M.E.R. and D.-S.P.) to ensure content integrity.

## 3. Results and Discussion

### 3.1. PRISMA Guideline 

The initial search in PubMed and Embase databases identified 290 records from the last 5 years, out of which 116 were duplicates and were excluded. A total of 106 studies with inadequate thematics were excluded after reading the title and abstract. Of the 68 remaining studies, 22 articles were excluded after reading the full text for not meeting the eligibility criteria. Following these exclusions, 46 were suitable for inclusion in the systematic review. The reference list includes five phytochemical studies; fourteen in vitro studies; nine in vivo studies; two studies both in vitro and in vivo; fourteen studies both phytochemical and in vitro; a phytochemical, in vitro, and in vivo study; and, respectively, a phytochemical, in silico, in vitro, and in vivo study. The flowchart of the review and each step performed in the selection process are shown in Figure 1. Table 1 shows the characteristics and the main findings of the studies included in the systematic review.

### 3.2. Publication Characteristics

Among the studies included in this review, 10.87% of the studies evaluated the phytochemical composition of oak galls (*n* = 5); 30.43% of the studies evaluated the effects of oak galls in vitro (*n* = 14), 19.57% in animal models or humans (*n* = 9), 4.35% both in vitro and in vivo (*n* = 2); 30.43% evaluated both the phytochemical composition and the in vitro effects (*n* = 14); 2.17% evaluated both the phytochemical composition and the in vitro and in vivo effects (*n* = 1), respectively; and 2.17% evaluated both the phytochemical composition and the in silico, in vitro, and in vivo effects (*n* = 1), respectively.

Regarding the country where the studies in this review were conducted, Malaysia dominated with 21.74% of the studies conducted on oak galls in the last 5 years (*n* = 10), followed by Iraq with 17.39% (*n* = 8), Iran with 15.22% (*n* = 7), China with 13.04% (*n* = 6), India with 8.70% (*n* = 4), Egypt and Turkey with 6.52% (*n* = 3), Saudi Arabia with 4.35% (*n* = 2), and Indonesia, Pakistan, and Thailand, respectively, with 2.17% *(n* = 1).

### 3.3. Phytochemicals Found in Quercus sp. Galls

The articles analyzed in our review revealed that the positive effect of oak galls, consisting of their numerous biological activities, can be attributed to the presence of various bioactive substances.

According to the findings, the composition of the metabolites found in the *Quercus* sp. galls showed great variation, both quantitatively and qualitatively, despite the fact that they all came from the same species.

Among the articles selected for this review, 39.13% of the studies examined the phytochemical composition of oak galls (*n* = 18), and 6.52% of the studies performed only the phytochemical screening *(n* = 3), while 8.70% of the studies targeted both the phytochemical screening and the study of the phytochemical composition (*n* = 4). Some studies analyzed the effects of oak galls but did not perform a phytochemical characterization (54.35%; *n* = 25).

Of all the studies included in the review, 36.96% investigated the main compounds responsible for the biological activities of oak galls, i.e., phenolic constituents, including phenolic acids and their esters, phenolic alcohols, hydroxyphenols, and dihydroxyphenols, respectively, and their derivatives, flavonoids, naphthodianthrones, prenylated phloroglucinol derivatives, coumarins and stilbenes, and also hydrolysable tannins—gallotannins and ellagitannins (*n* = 17). Only 13.04% of the studies examined other types of non-phenolic compounds present in oak galls, including lipid compounds, hydrocarbons, alcohols, carboxylic acids, ethers, esters, proteins, and elements (*n* = 6).

#### 3.3.1. Sample Preparation and Phenolic Compound Extraction

For phenolic identification research, it was essential to take into consideration the sample preparation. Due to the intricate nature of the majority of samples, the method employed for their preparation typically exerted a discernible influence on the outcomes of the entire extraction process. Several standard sample preparation methods, such as drying, homogenization, filtration, and grinding, were commonly employed prior to the extraction process [54].

Regarding the oak gall samples, their preparation before the extraction of phenolic compounds consisted of cleaning, drying, and grinding. The cleaning of the gall samples was performed with washing [8,43], some studies using tap water [19], and others boiling water [9]. The galls were air-dried at room temperature [19,43], in the shade [8,20] or in an oven at 40–45 °C for approximately 24 h [9,21]. For the coarse grinding of the plant material, either grinding the galls in a disc mill [51] or crushing the galls in a mortar with a pestle [8,43] was used. Grinding into fine particles was carried out using an electric grinder [21] or a vibrating-type ultrafine grinder [51]. To obtain a uniform powder, grinding was followed by sieving through sieves of different diameters [8,21,51]. The particle size of the powders subjected to extraction varied from 0.5 mm to <50 μm [9,21,43,51].

The solvent extraction method was commonly used to prepare crude extracts [54]. Phenolic compounds were extracted through the utilization of solvents with varying degrees of polarity, including methanol, ethanol, water, ethyl acetate, acetone, and/or their combinations [55]. In the present review, methanol was the solvent used for extraction in most of the phenolic composition studies (*n* = 7), followed by ethanol (*n* = 6), water (*n* = 5), and ethyl acetate (*n* = 2). Other solvents used were acetone (*n* = 1), n-butanol (*n* = 1), and mixtures, water/diethyl ether/ethyl acetate (*n* = 1) and diethyl ether/ethanol/water (*n* = 1). Among the conventional extraction techniques, maceration extraction [14,19,21,34,47], decoction technique [15], digestion technique [8,9,10,40,47], exhaustive serial extraction [15], soxhlet extraction [33], and reflux extraction were used [17,18,51].

Although the aim of an extraction process should be to ensure a maximum yield of active substances and of the highest quality, only a few of the studies included in the review aimed to optimize some parameters of the solvent extraction process, among the investigated variables being sample pre-treatment (particle size reduction), type of solvent, extraction method or extraction time, and temperature.

Reducing the particle size should increase the surface area available for mass transfer and increase the extraction yield [56]. Lu et al. [51] investigated the influence of a vibratory ultrafine grinding treatment on the physical and chemical properties and antioxidant activity of Turkish gall powder (TGP) with particle sizes >450, 400–250, 250–100, 100–50, and <50 μm, and they concluded that for the TGP extract with the smallest particle size (<50 μm), the highest gallic acid content (9.47 mg/g), methyl gallate content (34.78 mg/g), and ellagic acid content (0.79 mg/g) were obtained. Thus, reducing the particle size with ultrafine grinding facilitated the release of the three components from Turkish galls and consequently contributed to the increased DPPH, hydroxyl radical, and superoxide radical scavenging activities.

Regarding the type of solvent used to extract phenolic substances from natural sources, alcoholic solvents have been commonly used because they lead to a high yield of the total extract, although they are not highly selective for phenolics. In contrast, mixtures of alcohols and water were found to be more efficient in the extraction of phenolic constituents than the corresponding mono-component solvent system [56]. Thus, a study that aimed to evaluate the content of tannic acid, a well-known gallotannin, in different extracts of *Quercus* sp. galls using an HPLC analysis used for the extraction four different mixtures of solvents and water (96% ethanol, 80% ethanol, 70% acetone, and diethylether/ethanol/water mixture (25:3:1)), and two extraction techniques (maceration extraction and digestion technique), establishing that the highest amount of tannic acid (127.683 mg/g) was obtained in the 80% ethanolic extract obtained with maceration [47].

Comparing the effect of different solvents on the extraction efficiency of polyphenolic compounds, it was observed that the number of identified gallotannins varied from seven compounds identified in the case of extraction with ethyl acetate [16] or a mixture of solvents (water/diethyl ether/ethyl acetate) [17] to nine compounds in the extraction with ethanol [14], respectively, and thirteen compounds in the case of aqueous extraction [18], in all cases mass spectrometry (MS) was being used as the identification method.

In the case of phenolic acids, the number of representatives identified was higher when alcoholic solvents were used, than when the extraction solvent used was water. Thus, the extraction in methanol led to the identification of 11 phenolic acids by each of the two research teams led by Kılınçarslan Aksoy et al. [10,40]; the ethanolic extracts allowed the identification of 11 [14] and, respectively, 14 phenolic acids [21], while only 4 [18] and, respectively, 5 representatives [43] were identified in the aqueous extracts. The lowest extraction efficiency was observed when the solvents used were ethyl acetate [16] and, respectively, a mixture of solvents, water/diethyl ether/ethyl acetate [17], which led to the identification of only two phenolic acids.

The efficiency of solvents in the extraction of phenolic compounds varies, on the one hand, depending on the matrix, whether it is grassy or lignified (e.g., for the extraction of phenolics from hazelnut skin, maximum efficiency was obtained with 80% acetone [57] and 50% acetone for the walnut septum [58]), and, on the other hand, on the type of phenolic compounds, their polarity, and antioxidant activity being different, depending on the class of phenolic compounds. Some phenolic compounds are more polar, and they are more easily extracted in water, e.g., flavonoids; others are more easily extracted in alcohols or other less polar solvents. Depending on the phenolic compound composition of the plant, the extraction yields differ in different solvents.

Extraction temperature is another extraction parameter that plays a significant role in achieving an optimal quality of the extracted bioactive compounds because a high temperature can either increase the amount of extracted active compounds or cause their degradation. Using a conventional extraction method that was not used in the studies of this review, namely, the aqueous decoction method, a recent study investigated the effects of extraction temperatures (50, 75, and 100 °C) on the extracted tannin (tannic acid) content from the galls of *Q. infectoria* and on the antioxidant activity. The outcomes showed that the extraction temperatures had significant effects on the response variables (tannin content and antioxidant activity), the highest tannin concentration (2233.82 ± 1.311 mg/g) and the highest antioxidant activity (93.422 ± 0.256%) being obtained at the extraction temperature of 75 °C, this temperature being optimal for the hydrolysis of condensed tannins and the release of more active monomers [59].

In recent years, in addition to the conventional techniques used for the extraction of phenolic compounds from plant materials, unconventional extraction techniques, i.e., assisted extraction methods, such as those involving ultrasounds, microwaves, and pressurized/supercritical fluids, have also begun to be commonly used [54,60]. The innovative extraction techniques including supercritical fluid extraction [9] and ultrasound-assisted extraction via two types of the system, either classical ultrasonic-bath assisted extraction (CUBAE) or ultrasonic-probe assisted extraction (UPAE) [43], were also used in the reviewed studies.

Thus, in research that aimed to extract phenolic acids from oak galls using the UPAE method in the presence of ionic liquid, several variables were investigated on which the efficiency of the extraction of these compounds depends, namely, sonication time, extraction methods, solid-to-solvent ratio, type of solvent, and its concentration. The UPAE method was compared with the CUBAE method and the conventional aqueous extraction (CAE) method, with and without the presence of ionic liquid [43]. In contrast to the results obtained with the conventional extraction techniques (maceration and digestion) [47], the maximum amount of tannic acid (2430.48 mg/g) was extracted when the innovative UPAE extraction technique was used, in the presence of the ionic liquid [Bmim][Tf2N] [43].

A previous study investigated the effect of sonication time (from 1 to 12 h), solvent types (water and Hexadecyltrimethylammonium bromide (CTAB)), and solvent concentration (from 0.05 M to 0.2 M) on the extraction yield of gallic and tannic acids, extracted from *Q. infectoria* galls, using two ultrasound extraction systems (UPAE and CUBAE), and the results were compared with the conventional extraction system. The results showed that the UPAE extraction technique with 0.1 M CTAB as a solvent led to the maximum extraction yield of gallic acid and tannic acid (2155.77 mg/kg and 15,236.83 mg/kg, respectively) and shortened the extraction time (8 h), being more efficient than the CUBAE method and conventional extraction [61].

In the experiment by Purbowati et al. [9], the supercritical CO_2_ extraction method using methanol as a co-solvent led in the LC–MS/MS analysis of the extract to a more complete phenolic composition (27 phenolic compounds) and a higher amount compared to the extraction method without using a co-solvent (12 phenolic compounds). Methanol, with its polarity, favors the solubilization and extraction of more polar phenolic compounds, such as phenolic acids and flavonoids (Table 2), and thus improves the extraction yield. By changing the co-solvent concentration, the selectivity of the extraction process can be modulated. In another recent study, which also used the supercritical CO_2_ method with the addition of methanol as a co-solvent for the extraction of hydrolyzable tannins from *Q. infectoria* galls, the optimization of the extraction conditions was aimed at the extraction yield and the content of tannic acid and gallic acid. Thus, at the optimal values of the parameters (pressure, temperature, and mean particle size), optimal responses were achieved, such as an increased concentration of gallic acid (96.85 mg/g sample), but also of tannic acid (6149.71 mg/g sample), the latter being higher compared to the tannic acid concentrations reported in the studies selected from this review and obtained with other extraction methods (solvent extraction or UPAE method) [62].

Another work that aimed to identify and quantify the phenolic compounds from the extracts of the nutgall of Iraqian Aleppo oak (*Q. infectoria*) with LC-MS/MS used three different solvents and two extraction methods to obtain the extracts. The results showed that the extraction yield was strictly dependent on the nature of the solvents and extraction methods, methanol being the solvent that extracted the most components from the plant, followed by ethanol and water, respectively, and the microwave extraction technique proved to be much more efficient than the conventional one, considering the extraction yield [63].

Literature evidence suggests that these innovative extraction methods are preferred over conventional methods due to their numerous advantages, such as reduction in extraction time, temperature, organic solvent consumption, or reduction in toxic residues, as well as higher yields and improved experimental reproducibility [43,54].

#### 3.3.2. Separation and Characterization of Phenolic Compounds in *Quercus* sp. Galls

The extracts obtained by using the previously mentioned extraction techniques were complex products that needed to be separated because they contained a variety of natural components, as well as impurities. Separation is a purification technique, and it is frequently combined with characterization techniques to identify various molecules. The methods applied in phenolic compound separation include centrifugation, ultrafiltration, concentration of extracts, solvent separation methods, and chromatographic methods [60]. In this review, centrifugation [47,51] and concentration of the extracts [17,19,33,34,47], but also chromatographic methods [15,33,43], were used to separate the phenolic compounds present in the oak gall extracts.

Regarding the characterization of phenolic compounds, the identification of individual phenolic classes is usually performed with liquid chromatography (LC), gas chromatography (GC), or high-performance liquid chromatography (HPLC) and their detection using sensitive detectors [64].

LC assisted with mass spectrometry (MS) detection is an advanced analytical technique that, in recent years, has been used for the analysis of phenolic compounds due to its high sensitivity and selectivity [64]. Liquid chromatography with tandem mass spectrometry (LC–MS/MS) is considered one of the most reliable techniques for characterizing phenolic compounds [60]. In this review, LC-MS (*n* = 2) [16,17], LC-MS/MS (*n* = 3) [9,14,20], and liquid chromatography–electrospray ionization–tandem mass spectrometry (LC-ESI-MS/MS) techniques (*n* = 1) [18] were employed to assess the phytochemical profiles.

LC-MS is a useful tool in the metabolic profiling of plant samples, which has demonstrated its significant role in the identification, purification, and characterization of phenolic acids and flavonoids from the richest source of phenolic compounds with excellent antioxidant properties, namely, green leafy vegetables [65]. The LC-MS/MS method was also used in a study for the quantitative estimation of five phenolic acids, i.e., gallic acid, ellagic acid, corilaginic acid, caffeic acid, and syringic acid, and three flavonoids, respectively, i.e., rutin hydrate, quercetin, and morin hydrate, in the aqueous and hydroalcoholic extracts of *Q. infectoria* galls [66].

HPLC is a separation and characterization method, which can be combined with different detectors, such as an ultraviolet–visible (UV) and photodiode-array detector (PDA), to examine phenolic compounds [60]. HPLC coupled with PDA, also known as a diode-array detector (DAD), is the most useful and common method for analyzing the phenolic compounds in plants [64]. Even though LC-MS or LC-MS/MS are useful methods, the HPLC technique with UV detection is more accessible and successfully used in the quantification of phenolic compounds in plant extracts. Indeed, most of the reports in this review employed the HPLC-DAD/HPLC-PDA technique to identify phenolic compounds (*n* = 10) [8,10,15,21,33,34,40,43,47,51].

HPLC-DAD chromatographic separation was also used in a previous study to separate 13 phenolic acids and derivatives from galls, including hydroxybenzoic acids and hydroxycinnamic acids, among them gallic acid, 3,4-dihydroxybenzoic acid, syringic acid, and ellagic acid [67]. Phenolic acids were mainly detected using UV–visible, DAD, or fluorescence detectors [65]. The HPLC-UV technique was also utilized to detect the compounds gallic acid and 1, 2, 3, 4, 6-O-pentagalloyl glucose in a study that aimed to identify anticancer compounds through a PCA-constructed secondary metabolite map in Galla Chinensis and Galla Turcica gallnuts [68].

GC is considered an ideal method for the separation, identification, and quantification of some phenolic compounds in plants, such as tannins, flavonoids, and anthocyanins [64]. In recent years, due to its great selectivity and sensitivity in quantification, GC coupled with an MS detector has become increasingly common for analyzing complex compounds [60,64]. GC-MS was used for phytochemical characterization of *Q. infectoria* galls in one of the studies in this review [19]. In a previous study, Hussein et al. [69] also conducted the phytochemical screening of the methanolic dried galls’ extract of *Q. infectoria*, and the GC-MS analysis of the methanolic extract showed a highly complex profile containing twelve phytochemical compounds.

Thin-layer chromatography (TLC) is a relatively inexpensive chromatographic technique that can separate phenolic compounds in crude plant extracts and detect several substances on the same TLC plate in a relatively short amount of time [64]. This method was also applied in some works of the present review (*n* = 2) [15,33]. This technique was also employed by Ou et al., who developed a simple, rapid, and efficient TLC chromatographic method for the analysis and quantitative determination of ellagic acid, gallic acid, and methyl gallate in the galls of *Q. infectoria* Olivier. The conclusions showed that methyl gallate possessed the highest antioxidant efficacy, followed by gallic acid and ellagic acid, and the TLC-DPPH method could be exercised for the screening of antioxidant components [70].

Table 2 summarizes the studies that investigated the presence of phenolic compounds in oak galls and the related methodology.

#### 3.3.3. Phenolic Compounds of *Quercus* sp. Galls

Both simple phenolic compounds and polyphenols were identified in the galls collected from *Quercus* sp. In total, 67 phenolic compounds and their isomers or derivatives were described in the eligible studies.

Among the simple phenolic compounds, three subclasses were found in oak galls: simple phenolics, i.e., hydroxyphenols (catechol) and derivatives (2-allyl-5-t-butylhydroquinone), and dihydroxyphenols (pyrogallol) and derivatives (pyrocatechol); coumarins (coumarin); and phenolic acids.

In a previous study, pyrogallol was the major component extracted from *Q. infectoria* galls that displayed significant anti-*Candida* activity; however, due to the synergistic effect, the whole plant extract had a more potent antimicrobial activity compared to isolated phytomolecules [71].

From the subclass of phenolic acids, hydroxybenzoic acids (salicylic acid, *p*-hydroxybenzoic acid), dihydroxybenzoic acids (protocatechuic acid, gentisic acid) and their derivatives (vanillic acid), trihydroxybenzoic acids (gallic acid) and gallic acid derivatives (*m*-digallic acid, *p*-digallic acid, digallic methyl ester, digallic dimethyl ester, trigallic dimethyl ester, ellagic acid, syringic acid, 2-*O*-galloyl hydroxymalonic acid), tetrahydroxybenzoic acids (quinic acid), as well as hydroxycinnamic acids (cinnamic acid, *p*-coumaric acid, caffeic acid, ferulic acid, isoferulic acid) and their derivatives (chlorogenic acid, rosmarinic acid) were identified.

A survey that aimed to investigate the additional effects of active constituents from a *Q. infectoria* extract on staphylococcal cytoplasmic membrane function concluded that among the major components of the extract included in the study (ellagic, gallic, syringic, and tannic acids), only gallic acid and tannic acid, respectively, demonstrated good MIC⁄MBC values at the test concentrations and showed activity against methicillin-resistant *Staphylococcus aureus* [72]. Recent research demonstrated the anti-proliferative effects of two anticancer active compounds, tannic acid and gallic acid, extracted from *Q. infectoria* galls, on the human glioblastoma multiforme cell line (DBTRG-05MG) [73]. A previous experiment revealed that the ellagic acid glycoside, quercoside, isolated from the ethanolic extract of *Q. infectoria* Olivier galls, possessed nitric oxide and superoxide inhibiting activity in murine macrophages [74].

**Figure 2:**
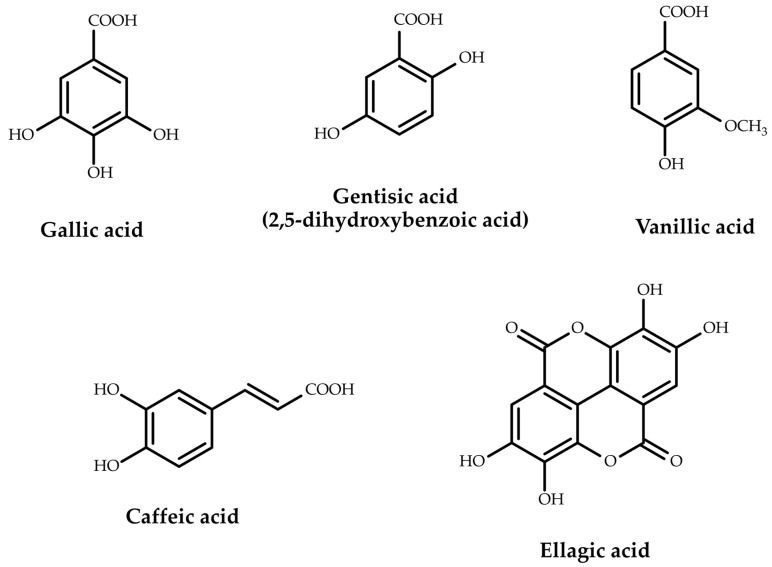
Representative phenolic acids found in oak galls.

The structures of the representative phenolic acids are presented in Figure 2.

**Figure 3:**
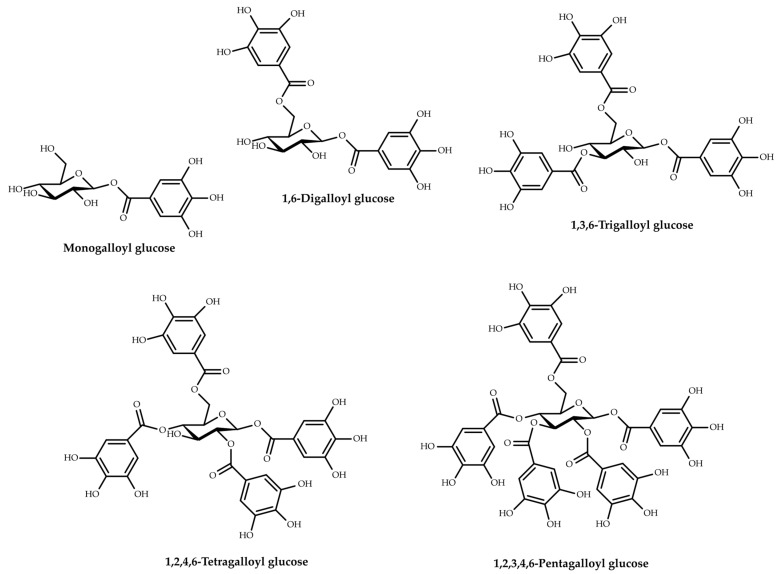
Key gallotannins reported in oak galls.

The polyphenolic compounds analyzed in the studies belong to two large polyphenol subclasses, flavonoids and tannins. Four types of flavonoids were identified, including flavones (luteolin, chrysin, apigenin, 7-hydroxyflavone) and their derivatives (apigetrin, lucenin 2), flavonols (kaempferol, myricetin, quercetin, fisetin) and their derivatives (quercitrin, rhamnetin, hyperoside, astragalin, rutin), flavanones (naringenin, hesperetin) and their derivatives (hesperidin, luteolin-7-glucoside, naringin), and flavan-3-ols (catechin, epicatechin). Among the tannins, only the hydrolyzable ones were identified, i.e., gallotannins and ellagitannins. The gallotannins were mainly represented by tannic acid (syn. gallotannin), but also by methyl gallate and mono-, di-, tri-, tetra-, penta-, hexa-, and hepta-galloyl-glucose (Figure 3).

A recent investigation that tested the antioxidant activity of *Q. infectoria* galls, on both chemical and biological models, argued that the strong antioxidant activity of the ethanolic extract (scavenging of DPPH and •OH radicals, Fe^2+^ chelation, inhibition of lipid peroxidation, and protection of macrophages from oxidative damage induced with tertiary butyl hydroperoxide) could be attributed at least partially to the tannic acid that constituted a major proportion of the extract (19.925%), but also to gallic acid (8.75%) [75]. A former work exposed the inhibitory activity of hexagalloyl glucose isolated from a *Q. infectoria* gall methanol extract against alpha-glycosidases, which was comparable to the hypoglycemic agent acarbose [76]. Another investigation that studied the inhibition effectiveness and specificity of Aleppo tannin (gallotannin), isolated from the gallnut of the Aleppo oak, on human salivary amylase confirmed that it was a more efficient amylase inhibitor than tannin with a quinic acid core [77].

The ellagitannins were identified in a single study, being represented by galloyl-HHDP-glucose and pedunculagin.

Other phenolic compounds were less analyzed in the included studies, such as benzaldehydes (vanillin), naphthodianthrones (pseudohypericin, hypericin), and prenylated phloroglucinol derivatives (hyperforin), which were identified in one study, and phenolic alcohols (3-hydroxytyrosol) and stilbenes (resveratrol), which were quantified also only in one experiment among the forty-six selected.

Despite the great diversity of structural classes and subclasses assessed in oak galls, the predominant phenolic subclasses were represented by phenolic acids, gallotannins, and flavonoids. Of the analyzed studies, over 28% identified phenolic acids (*n* = 13), while gallotannins and flavonoids were found in 26.09% (*n* = 12) and 17.39% (*n* = 8), respectively.

In quantitative analyses, gallic acid was evaluated in most of the phenolic composition studies (*n* = 7), followed by ellagic acid (*n* = 6) and caffeic acid (*n* = 5), these three representatives also proving to be the most abundant in oak galls compared to the rest of the quantified phenolic acids.

Among the phenolic acids, gallic acid is recognized as the most prevalent hydroxybenzoic acid, being abundant both in natural sources (oak gallnuts/leaves/bark/acorns, pomegranate root bark, berry/tea leaves, many fruits and vegetables), as well as in processed beverages (red wine and green tea) [78,79,80]. Significant amounts of gallic acid were reported in oak galls, both in the case of extraction with a conventional technique (291 mg/g dry weight (dw)) [8] and with ultrasonic-probe assisted extraction (UPAE) (130.76 mg/g dw) [43].

Ellagic acid is a dimeric gallic acid derivative, widely present in fruits (pomegranate, mango, grapes), berries (blackberry, raspberry, blueberry, cranberry, and strawberry), nuts (walnuts, pecans, chestnuts, almonds), seeds, dry fruits, and some types of honey, but also in herbs, roots, and alcoholic beverages matured in oak wooden barrels [78,81]. In oak galls, the highest amounts of ellagic acid (261,997.718 and 187,696.132 μg/g dw, respectively) were reported in two studies by Kılınçarslan Aksoy et al. [10,40]. In the study conducted by Shendge and Kamalapurkar [8], the concentration of ellagic acid (131 mg/g dw) was also much higher than in three other studies (0.64–33.44 mg/g dw).

The results of previous reports confirmed that phenolic acids were widely distributed in all oak matrices, gallic acid being found in leaves and acorns, ellagic acid in leaves, bark, seeds, and wood, and caffeic acid in wood of several species of *Quercus* [79]. In a study that evaluated the phenolic composition of oak galls, gallic acid and ellagic acid were found to be the most abundant phenolic components in aqueous and hydroalcoholic extracts. For both, gallic acid and ellagic acid, the concentration in the aqueous extract (106,711.25 ± 951.25 μg/g dw and, respectively, 5105.03 ± 102.34 μg/g dw) was higher than in the hydroalcoholic extract (84,613.34 ± 589.12 μg/g dw and 3522.31 ± 82.36 μg/g dw, respectively) [66]. In contrast, another experiment reported a much lower content for gallic acid (3724.12 μg/g dw) in the methanolic extract of *Q. infectoria* nutgalls [63].

Caffeic acid, a hydroxycinnamic acid derivative, is found in various natural sources including olives, berries, potatoes, and carrots, with coffee beans being particularly rich in this compound [78]. Its concentration in oak galls varied widely among the five studies included in the review, between 0.50 mg/g dw and 589.041 mg/g dw [10,21,34,40,43]. A recent work reported a lower caffeic acid content in oak galls, 0.07 ± 0.01 μg/g dw in the aqueous extract and 1.70 ± 0.16 μg/g dw in the hydroalcoholic extract [66].

Gallotannins, considered the simplest hydrolyzable tannins, are formed by gallic acid molecules bound to a central d-glucose with ester bonds (Figure 3) [82]. Tannic acid (penta-*m*-digalloyl glucose) is composed of a central glucose esterified to all five hydroxyl moieties with two molecules of gallic acid, totaling ten galloyl groups [83]. The concentration of tannic acid in oak galls was determined in five studies and varied depending on the type of solvent and extraction method used. The lowest amounts were reported in the case of maceration with a mixture of solvents (diethylether/ethanol/water (25:3:1)) (0.016–0.112 mg/g dw) [47], while the highest amounts were obtained using the UPAE method, 2287.90 mg/g dw in the presence of ionic liquid and 776.75–1556.26 mg/g dw in the absence of ionic liquid [43]. Another recent survey [59] reported a concentration of tannic acid (2233.82 ± 1.311 mg/g) in the aqueous decoction of *Q. infectoria* (Manjakani) galls, which was much higher than the amounts obtained with conventional extraction methods in the works of this review. The same was true for Mohd-Nasir et al. [62], who obtained a higher amount of tannic acid (6149.69 mg/g) in *Q. infectoria* galls’ extracts in the case of supercritical CO_2_ extraction versus the results mentioned in our review for unconventional extraction techniques. Methyl gallate was quantified in a single study, with reported concentrations varying between 26.07 and 34.78 mg/g, depending on the size of the Turkish gall powder particles [51].

Flavonoids are bioactive polyphenolic phytochemicals consisting of a 15-carbon (C6–C3–C6) skeleton that is composed of two benzene rings (C6) and a 3-carbon (C3) linking chain [54,84]. They are abundant compounds in nature, being present in most plants and in numerous foods, such as fruits, vegetables, legumes, nuts, medicinal plants, tea, chocolate, or red wine [54,85,86]. In oak galls, the most prevalent flavonoid compound was quercetin, quantified in five phenolic composition studies [10,21,34,40,43], with the highest amount reported in the work of Mohammadzadeh et al. (5.00 mg/g dw) [34]. Other assays reported lower concentrations for quercetin, namely, an amount of 6.36 ± 0.81 μg/g dw in the aqueous extract, 0.38 ± 0.05 μg/g dw in the hydroalcoholic extract [66], and, respectively, 3.7597 μg/g dw in the methanolic extract [63]. Among the flavonoids detected in significant amounts, the highest concentrations were reported for the two flavan-3-ols, epicatechin (171,497.57 μg/g dw) [10] and catechin (15,622.42 μg/g dw) [21], respectively, but also for naringin (19,097.058 μg/g dw) [10] and rutin (10.72 mg/g dw μg/g dw) [34]. Lower concentrations were reported for five other flavonoids, i.e., myricetin (0.05–0.55 mg/g dw), apigenin (0.01–0.09 mg/g dw) [43], quercitrin (89.82 μg/g dw), hesperetin (4.66 μg/g dw), and 7-hydroxyflavone (3.5 μg/g dw) [21], each of them being quantified in a single phenolic composition study. A previous experiment performed the quantitative analysis of several flavonoid compounds in the methanolic extract of *Q. infectoria* nutgalls, compounds that in the studies of this review were only identified. Thus, the authors quantified hyperoside (44,534 μg/g dw), hesperidin (24.788 μg/g dw), kaempferol (0.6318 μg/g dw), luteolin (0.1357 μg/g dw), naringenin (0.110 μg/g dw), rhamnetin (0.0639 μg/g dw), and fisetin (0.00957 μg/g dw). On the other hand, for the rest of the flavonoids quantified, namely, rutin (2.4745 μg/g dw), myricetin (0.54704 μg/g dw), apigenin (0.0701 μg/g dw), and hesperetin (0.0374 μg/g dw), the results were lower compared to those reported in our review [63].

Despite such a diverse and rich phenolic profile of this plant matrix, studies have shown that the positive activity results could be attributed to the main constituents of *Q. infectoria* galls, including tannic acid constituents (50–70%), especially tannic acid and gallotannins containing mixtures of polygalloyl groups, and gallic acid, which represents 2–4% of the total compounds, but also to other minor components, such as ellagic acid and syringic acid [7,72,73,75,77].

#### 3.3.4. Non-Phenolic Compounds of *Quercus* sp. Galls

The largest diversity of non-phenolic molecules, i.e., a total of 34 compounds and nine elements, was reported in the study of Jalill [50], while five other studies identified 16 other non-phenolic compounds [9,19,21,34,43]. Terpenes and terpenoids were present in a larger number, i.e., 17 compounds, followed by lipid compounds, hydrocarbons, and carboxylic acids. The presence of lipid compounds, i.e., fatty acids, fatty amides, and fatty aldehydes, was reported in two of the studies included in the review. The same studies identified aliphatic alcohols in the oak galls [19,50].

Among the six reports that investigated the presence of non-phenolic compounds in oak galls, only three studies performed their quantitative analysis. Carboxylic acids were detected in significant amounts, the highest concentration being reported for malic acid (79.28 mg/g dw) [43], followed by aconitic acid (20.37 mg/g dw) [43] and benzoic acid (9.25 mg/g dw) [34]. Also, Tayel et al. [21] obtained a significant concentration of caffeine (21.676 mg/g dw).

Other surveys that analyzed the non-phenolic phytochemical composition of *Q. infectoria* galls mainly investigated the volatile and lipid composition, with GC-MS. Thus, a recent study characterized 29 substances in the volatile essential oil of *Q. infectoria*, the majority component being (Z)-anethole (28.55%) [87]. This was also identified in Jalill’s experiment [50], along with three other main components, pentadecanolide (26.44%), diethyl phthalate (6.46%), and acetoin (5.66%) [87]. In another study, Hussein et al. [69] identified 12 bioactive compounds in the methanolic dried galls’ extract of *Q. infectoria*, including phytosterols, monoterpenes, or pteridines, some of them being known for their antimicrobial, anti-inflammatory, and antitumor activities; anti-psychotic, mood-stabilizer, and anti-parasite actions; as well as estrogenic, progesterogenic, and anti-infective effects.

Table 3 shows the non-phenolic compounds identified and/or quantified in the oak galls.

### 3.4. Biological Activities

#### 3.4.1. In Vitro Activity

##### Antioxidant and Anti-Inflammatory Activities

The antioxidant properties of oak galls demonstrated in both in vitro and in vivo studies [10,12,15,27,30,33,34,40] could be attributed to their phytochemical profile. Many of their components, such as phenolic acids, flavonoids, and hydrolyzable tannins, but also hydroxyphenols, coumarins, phenolic aldehydes, naphthodianthrones, acyl-phloroglucinols, and phenolic alcohols, showed antioxidant effects through direct free-radical scavenging action or indirect action. Previously, Kaur et al. [75] reported that the polyphenols present in a *Q. infectoria* gall extract possessed a potent reducing power, scavenging free radicals, such as DPPH (IC_50_~0.5 μg/mL), ABTS (IC_50_~1 μg/mL), and hydroxyl (*OH) radicals (IC_50_~6 μg/mL).

The mechanisms were by complexing some metals involved in the oxidative stress induction or by activating cellular signaling pathways associated with cytoprotective mechanisms: up-regulation of the Nrf2/ARE pathway and down-regulation of the NF-κB transcription factor pathway, followed by reduction in inflammatory processes [88]. Some of these metabolites including flavonoids could induce antioxidant and anti-inflammatory responses through scavenging free radicals, up-regulating HO-1 expression, inhibiting the COX-2 and 5-LOX proinflammatory signaling pathways, or modulating the function stabilization of the intestinal barrier, thus contributing to the intestinal wall and blood–brain barrier integrity via the gut–brain axis [81].

Zang et al. [17] identified nine category constituents including phenolic acids and gallotannins in Turkish galls. Among them, methyl gallate, digallic acid, di-*O*-galloyl-β-d-glucose, and tri-*O*-galloyl-β-d-glucose mainly contributed to the anti-inflammatory activity via suppressing the NO, IL-6, and TNF-α production. Similar compounds including phenolic acids (cinnamic acid, *p*-coumaric acid, ferulic acid) and gallotannins (digalloylglucose), found in other plant matrices, demonstrated active roles against oxidative stress and type 2 diabetes [89]. However, the presence of galls on leaves of *Q. robur* had a negative effect on cell membrane integrity and the antioxidant potential of the host plant [90].

Recently, the aqueous extract of *Q. infectoria* galls was suggested to have the potential for augmenting immunomodulatory activity and modulate the innate immune response through cellular-mediated mechanisms [36]. Thus, in gall-extract-treated murine macrophage (J774A.1) cells compared to untreated cells, the phagocytosis increased, while the NO production decreased in a dose-dependent manner. Moreover, the extract lowered IL-4, IL-6, and IL-12 gene expression and improved the output of anti-inflammatory cytokine IL-13, which can inhibit proinflammatory cytokine production in vitro. Previous studies have shown that a *Q. infectoria* gall extract could suppress oxidative stress and inflammation in murine bone-marrow-derived macrophages by inhibiting the Set-7/NF-κB pathway, therefore controlling chronic inflammation associated with several disorders including age-related diseases [91].

##### Antimicrobial Activity

Extracts of the *Q. infectoria* gall were revealed to have broad-spectrum in vitro antimicrobial activity. Our review assayed various studies (Table 1) that determined the antimicrobial activity of a *Q. infectoria* gall extract against pathogenic organisms and evaluated the morphological changes of extract-treated cells.

A *Q. infectoria* gall extract possessed efficient antimicrobial activity against *Streptococcus mutans*, *S. sobrinus*, and *Candida albicans* [11]. As this activity was synergistically enhanced in the presence of a *Scrophularia striata* extract, the two extracts may be used together for preparing dental products with anticariogenic potential.

A further analysis revealed that a *Q. infectoria* gall extract showed antimicrobial activity against *Staphylococcus aureus*, *Escherichia coli*, *Pseudomonas aeruginosa*, *Salmonella enterica* serovar Typhimurium, and *C. albicans*. Immersion in a 1% gall extract solution sharply reduced eggshell microbial contamination, while *E. coli* and *S. aureus* were completely suppressed after 60 min of immersion. The investigation revealed that gall extracts might be suggested as natural and effective disinfectants [21].

Similarly, the hydroalcoholic extract of *Q. infectoria* galls manifested high antimicrobial activity against *E. coli*, *S. aureus*, *S. epidermidis*, and *Klebsiella pneumonia*, as well as good antioxidant capacity due to the presence of polyphenols, especially gallic acid, rutin, quercetin, benzoic acid, and caffeic acid [34]. In addition, an ethanolic extract of *Q. infectoria* galls exhibited inhibitory and bactericidal effects on strains of *E. coli* with related antibacterial mechanisms from disruption of the outer wall and cytoplasmic membranes to loss of bacterial cellular integrity [92].

Additionally, Nair et al. [19] corroborated the antibacterial effect of *Q. infectoria* galls. At a dose rate of 50 mg/mL, the methanolic extract manifested a complete bactericidal effect on *S*. *enterica* ser. Typhi and *S*. *enterica* ser. Enteritidis, while at lower concentrations, had a significant bacteriostatic effect. At the same time, the antibacterial effect obtained from the combination of a *Q. infectoria* gall extract and methanolic extract from fruits of *Phyllanthus emblica,* rich in ellagitannins, was synergic, greater than the sum of the individual effects (*p* < 0.001) [19].

Yet another study revealed that the ethanol extract of *Q. infectoria* galls inhibited the growth of all the bacterial strains at a concentration of 1000 μg/mL and, in combination with ceftazidime, exhibited a strong synergistic activity on *P. aeruginosa* and *E. coli* [93].

A degree of novelty presented in our research was the use of oak galls’ extracts to prepare metal nanoparticles. Based on the fact that metal nanoparticles have good antimicrobial activity, several studies analyzed the effects of such treatments. The production of these metal nanoparticles employed green synthesis methods using an extract of *Q. infectoria* galls as a reducing and capping agent. Silver nanoparticles (AgNPs) and a *Q. infectoria* gall extract inhibited the growth of *P. aeruginosa* [49]. Silver nanoparticles are well known for antibacterial and immunostimulant activities [94]. In addition, a thermosensitive antibacterial gel from a *Q. infectoria* gall aqueous extract and AgNPs for the treatment of mouth ulcers and gum disorder were developed [8]. Bioactive compounds of oak galls, such as tannic, gallic, and ellagic acids, in addition to the nanoparticles that can penetrate the cell membrane and prevent the replication via interfering with bacterial DNA, showed in vitro activity against *P. aeruginosa*, *S. aureus*, and *E. coli*. The formulated gel, with antibacterial activity greater than the commercial gel containing only tannic acid, may be used as an internal topical in oral infectious disorders.

These results were consistent with other findings showing that the gall extract and AgNPs revealed excellent antioxidant capacity and antibacterial activity against *Klebsiella pneumonia* and *Enterococcus faecalis*, besides *P. aeruginosa* and *S. aureus*. Still, AgNPs significantly exhibited more antibacterial activities compared to the galls’ extract, with the highest antibacterial activity against *K. pneumonia*. Furthermore, both treatments exposed anticancer activity against human breast cancer cells (MCF-7); yet again, AgNPs exhibited stronger cytotoxic activity [27].

The antibacterial activity of a *Q. infectoria* gall extract and copper oxide nanoparticles (CuONPs) was evaluated against two Gram-positive bacteria and four Gram-negative bacteria, including *P. aeruginosa* and *E. coli*. This study, in which *Q. infectoria* galls were used for the first time to synthesize CuONPs, concluded that both treatments showed good antibacterial activity against Gram-positive and Gram-negative bacteria, but CuONPs significantly displayed more antibacterial activity compared to the oak gall extract [26]. Moreover, the extract of the *Q. infectoria* gall combined with a *Calendula officinalis* flower extract and CuONPs demonstrated considerable antibacterial function and significant wound-healing potentials [24].

*P. aeruginosa*, one of the most virulent Gram-negative bacterial pathogens in humans, causes many acute and chronic infections through a plethora of cytotoxins [95]. Ahmed and Salih confirmed the antibacterial activity of *Q. infectoria* gall extracts against *P. aeruginosa* [20]. This activity involved two mechanisms, either a direct growth inhibitory effect or the down-regulation of virulence-regulator genes. The potential ability to reduce the expression of these genes could be a valuable prophylactic and therapeutic use of oak gall extracts.

The pathogenicity of *Helicobacter pylori* can also be altered with *Q. infectoria* gall extracts. This pathogenic bacterium may be found in human gastric mucosa and can cause chronic stomach inflammation, peptic ulcer, or gastric adenocarcinoma. Attia et al. [14] evaluated the action of an oak gall extract and zinc oxide nanoparticles based on a *Q. infectoria* gall extract (Qi-ZnONPs) against *H. pylori*. Although both treatments exhibited moderate antibacterial activity, the Qi-ZnONPs displayed greater inhibition (98.4%) compared to amoxicillin (93.2%) and clarithromycin (90.7%). Moreover, the study concludes that the combination of Qi-ZnONPs and amoxicillin (4:1) is a potential candidate for an effective anti-*H. pylori* drug.

Ethanol and water extracts of *Q. infectoria* galls also demonstrated strong bacteriostatic activity against *Vibrio parahaemolyticus* and antibacterial efficacy against all bacterial strains. Besides these effects, an herbal formulation containing *Nigella sativa* seeds, *Piper retrofractum* fruit, *Punica granatum* pericarp, and *Q. infectoria* galls reduced the swarming motility of *E. coli* and inhibited biofilm production by *S. aureus* [41]. Another experiment proved that a methanolic extract of oak galls was more effective than a water extract against *S. sanguis*, *S. aureus*, *S. mutans*, and *S. salivarius* [96].

Moreover, a *Q. infectoria* gall extract combined with cetrimonium bromide displayed efficacy in the removal of *S. enterica* ser. Typhimurium biofilm, suggesting an alternative to remove biofilm from food contact surfaces in the household and food industry [97]. Biofilm defends bacteria from the surrounding environment, including antibiotic, antiseptic, and chemotherapeutic treatments. Periodontal diseases and dental caries are biofilm-mediated and are major public health concerns [98]. *Q. infectoria* gall extracts disclosed significant antibiofilm (92%) and antibacterial (19.00 ± 7.07 mm) activities against *Rothia dentocariosa*, a Gram-positive bacterial pathogen responsible for causing dental caries through biofilm formation [46]. Likewise, oak gall extracts showed antimicrobial activity against other oral bacteria. Among tested bacteria, the extract showed good antibacterial activity and ability to reduce biofilm against *S. aureus* and *S. mutans*, respectively [39]. An early report also exposed that *Q. infectoria* gall extracts had significant (*p* < 0.05) biofilm removal activity and antibacterial effects against *S. mutans* [99]. Thus, the oak galls may be considered preventing therapeutic agents of biofilm formation by oral pathogens.

The hydroalcoholic extract of *Q. infectoria* galls was also evaluated on *Aggregatibacter actinomycetemcomitans*, a bacterium associated with aggressive forms of periodontitis [100]. This in vitro study concluded that a hydroalcoholic extract of *Q. infectoria* galls may be used in mouthwashes to alter periodontal biofilm. Similarly, methanol and acetone extracts of *Q. infectoria* galls exhibited antibacterial activity against two Gram-positive (*S. mutans* and *S. salivarius*) and two Gram-negative bacteria (*Porphyromonas gingivalis* and *Fusobacterium nucleatum*) known to cause dental caries and periodontitis [101].

In a recent review, Taib et al. [102] stated that *Q. infectoria* galls possessed astringent, antiseptic, anti-inflammatory, and cicatrizing properties. Indeed, due to the fact that *Q. infectoria* galls contain large amounts of gallotannins and other bioactive components that have an astringent action on vessels and tissues, an oak gall extract could be used in preparations used to inhibit the growth of oral bacteria, with therapeutic effects in patients with gingivitis and bacterial plaque [8,37], including in the treatment of periodontitis, a pathology that frequently affects the elderly and/or patients with aging-related pathologies [103].

The antimicrobial action of a *Q. infectoria* gall extract was applied against skin pathogens with *S. aureus* strains being more sensitive than *C. albicans* strains [53]. Previously, the extracts of the *Q. infectoria* gall exhibited promising in vitro antibacterial activities, especially against Gram-positive bacteria including *S. aureus* [104], and displayed anti-*Candida* activity and could treat yeast infections caused by *Candida* species [71].

The bioactive compounds obtained from *Q. infectoria* galls also demonstrated antifungal activity against *Penicillium expansum* and *Aspergillus flavus* [25]. Both these pathogenic fungi produce mycotoxins, which can be toxic to humans. *P. expansum* produces patulin, a neurotoxic metabolite particularly for children [105], while *Aspergillus* sp. initiates aspergillosis, an infection usually of the lungs that may compromise the immune system and cause complications in the respiratory disorder population [106].

The results of a broth microdilution assay confirmed that the aqueous *Q. infectoria* gall extract displayed antimicrobial inhibition and killing activity against two pathogenic *Leptospira interrogans* isolates, therefore showing potential in the treatment of leptospirosis [52].

The reduced efficacy of the antimalarial medicines requires the need to develop new drugs that can target *Plasmodium falciparum*, the parasite causing malaria, one of the leading causes of death worldwide. Two experiments reported interesting in vitro antimalarial effects of oak gall extracts. Thus, the acetone and methanol extracts of *Q. infectoria* galls displayed promising antimalarial activity (IC_50_ = 5.85 ± 1.64 and 10.31 ± 1.90 μg/mL, respectively), while ethanol and aqueous extracts showed low activity [42]. Furthermore, acetone extract treatment significantly (*p* < 0.001) changed the pH of the digestive vacuole of the malaria parasite, *P. falciparum* [35]. New findings confirmed these results. Hence, ellagic acid, the phenolic compound found in oak galls, presented strong antimalarial activity similar to a standard drug, artemisinin, while the pH of the digestive vacuole of ellagic-acid-treated parasites was significantly altered (pH = 6.11 to 6.74, *p* < 0.001) in a concentration-dependent manner versus untreated parasites [107].

Considering these outcomes, the extract of *Q. infectoria* galls is a promising antimalarial treatment and could be used as a primary substance in treating different microbial infections and oxidative-stress-related diseases.

##### Anticancer Activity

Despite advances in treatment strategies, cancer statistic data show that the prevalence of cancer continues to rise worldwide. Due to the fact that conventional cancer treatments manifest low cure rates and numerous adverse effects, many cancer therapy strategies have lately included natural products, usually well tolerated even at high dosages, that can sensitize cancer cells, inhibit tumor growth and proliferation, and induce cell cycle arrest and apoptosis, thus representing a promising approach in the therapy of cancer. Several studies that assessed gall extracts reached outcomes consistent with these findings (Table 1).

Bioactive compounds from a water extract of *Q. infectoria* galls produced by *Cynips gallae tinctoriae* wasps contributed to the cytotoxic effect on colorectal cancer (CRC) cells. This cytotoxicity was related to the intracellular ROS accumulation, which triggered cancer cell growth limitation and autophagic cell death via inhibiting the AKT/mTOR signaling pathway. In addition, the gall extract significantly suppressed the epithelial mesenchymal transition (EMT) process known to be involved in tumorigenesis and migration of cancer cells. In addition, the activated extracellular signal-regulated kinase (Erk) signaling pathway promoted the autophagic CRC cell death [45].

A recent survey also explored the cytotoxic effects of galls of *Q. brantii*. The results showed that the extract at a concentration of 0.05 mg/mL significantly (*p* < 0.001) increased cytotoxicity, ROS formation, lipid peroxidation, and cytochrome-c release in A375 and SK-MEL-3 melanoma versus AGO-1522 normal human fibroblast cell lines [108].

A further analysis revealed the potent cytotoxic activity of a *Q. infectoria* gall extract against cervical cancer (HeLa) cells (IC_50_ = 6.33 ± 0.33 μg/mL) regulated with apoptotic cell death characterized by chromatin and nuclear condensation, DNA fragmentation, as well as apoptotic body formation [44]. Moreover, the *Q. infectoria* gall extract was shown to induce HeLa cell apoptosis via activation of caspase-8 and caspase-9 [28]. Ismail et al. [32] also demonstrated the cytotoxicity of *Q. infectoria* gall extracts on HeLa cells. The cancerous cells experienced apoptosis in response to the treatment, which was noticed in annexin V/PI staining and in acridine orange and propidium iodide (AO/PI) stained cells compared to the control (*p* < 0.05). These studies indicated that oak gall extracts significantly inhibited HeLa cell growth via apoptosis induction.

In the study of Jalill [50], all concentrations of *Q. infectoria* gall extracts decreased the mouse mammary carcinoma cell line, with IC_50_ = 0.2 mg/mL. Volatile compounds, such as eucalyptol and eugenol, found in gall extracts could be responsible for this activity. Eucalyptol and eugenol, known antioxidants [109], could suppress production of α-TNF, interleukin-1β, and leukotrienes, and inhibit human cancer cell proliferation through cell cycle arrest and autophagic and apoptotic effects [110,111].

Kilincarslan Aksoy’s research team analyzed the gall of *Andricus*, a genus of oak gall wasps. According to the outcomes, both *A. tomentosus* and *A. sternlichti* gall extracts contained important amounts of phenolics, flavonoids, and tannins associated with antioxidant, cytotoxic, and antiproliferative activities [10,40].

The regulation of the immune system is essential for prevention and treatment of infection, autoimmune diseases, and cancer.

Concomitantly, Kamarudin et al. [15] reported that specific active constituents of *Q. infectoria* galls have the potential to inhibit glioblastoma multiforme (GBM), a highly invasive stage IV malignant brain tumor. In this experiment, a two-phase system consisting of aqueous soxhlet extraction and methanolic enrichment fractionation was utilized to extract gallotannin, an anticancer component. This optimized system successfully produced a powerful fraction (F4) with around 71% gallotannin that had significantly higher antioxidant activities compared to its crude extract and to a commercial synthetic pure gallotannin. Related to its content and higher antioxidant property, the F4 was also established to better suppress GBM cell growth compared to the gall crude extract and pure gallotannin. Interestingly, the inhibitory capacity exerted with the F4 fraction on GBM cells was comparable to the effects of two clinically used chemo-drugs, Tamoxifen and Temozolomide, signaling the high efficiency of an enriched fraction of a *Q. infectoria* gall extract in fighting cancer cells in vitro.

#### 3.4.2. In Vivo Activity

Recent results revealed that the phytochemical bioactive molecules (gallotannins, gallic and elagic acids) of oak galls might be responsible for the diverse biological in vivo activities including anti-inflammatory, antioxidant, and antimicrobial properties, or anticancer potential (Table 1).

Ulcerative colitis (UC) is an inflammatory disease that belongs to the inflammatory bowel disease group describing chronic inflammatory conditions of the gastrointestinal tract and occurring from the proximal to the distal ends of the colon. Its etiology is not well defined, one possible cause being the proinflammatory cytokines that initiate an inflammatory event. Since the recommended anti-UC medications have only modest therapeutic effects, which could be associated with serious side effects, alternative therapeutic strategies with no toxicity have recently been explored.

According to the outcomes of an animal investigation, a rich fraction of a *Q. infectoria* gall extract, which included methyl gallate, digallic acid, di-*O*-galloyl-β-d-glucose, and tri-*O*-galloyl-β-d-glucose, had protective effects on the colon length of UC mice and ameliorated colon shortening, one of the parameters in the assessment of colonic inflammation [17]. The treatment exposed antioxidant and anti-inflammatory potential, significantly decreasing IL-1β, IL-6, TNF-α, ICAM-1, and TLR4 levels and inhibiting the NF-κB signaling pathway. Previously, Khanavi et al. [112] showed that the extract of the *Q. brantii* gall exerted an antioxidant effect by lowering the levels of cellular lipid peroxidation, and anti-inflammatory capacity via decreasing TNF-α and IL-1β levels, all biochemical and pathological biomarkers of UC.

A further analysis revealed that microcapsules of gallotannins isolated from *Q. infectoria* galls combined with iron (III) displayed anti-inflammatory effects in Kunming mice with induced UC [16]. The bioactive components were prone to attach to the surface of the inflamed colon epithelium, inhibit the plasma levels of TNF-α and IL-1β, and alleviate UC symptoms.

The gut microbiota and the balance between beneficial and pathogenic bacteria have a strong influence in many disease processes. The dysbiosis of the gut microbiome is a key pathogenetic mechanism, and the pathogenic bacteria in the UC animal intestinal tract were correlated with proinflammatory factors, while beneficial bacteria were linked with anti-inflammatory markers [113]. Several studies exposed that plant bioactive components could modulate the composition of intestinal microorganisms by stimulating beneficial bacteria and reducing pathogenic bacteria, thus promoting the expression of tight junction proteins, such as occludin and zonula occludens-1, in order to conserve the intestinal mucosal barrier function and prevent UC [114]. The experiment of Yu et al. [48], based on the idea that plant extracts might treat UC via intestinal flora modulation, was in line with the above studies. The treatment with extracts of *Q. infectoria* galls in UC mice reduced harmful bacteria, such as *Helicobacter*, *Bilophila*, and *Acinetobacter*, while the levels of SCFA-producing bacteria (e.g., *Bacteriodes*, *Allobaculum*, *Blautia*, *Butyricimonas*) and anti-inflammatory bacteria, *Lactococcus and Bifidobacterium*, were significantly increased, these results emphasizing the modulation of intestinal flora as another mechanism of *Q. infectoria* galls in treating UC.

Taken together, the preceding outcomes highlight that oak galls could efficiently modify UC inflammatory mediators and pathological markers, and, hence, might be promising natural agents in the management of UC.

Diabetes mellitus, an age-related chronic metabolic disorder characterized by hyperglycemia, polyuria, and polyphagia, leads to secondary pathophysiological dysfunction in various tissues [115]. Diabetes mellitus and thyroid diseases are two endocrine metabolic disorders that tend to coexist in humans, since thyroid hormones regulate the insulin secretion of pancreatic beta cells and regulate glucose homeostasis [116]. Furthermore, diabetes mellitus creates male infertility via an increase in ROS levels and a cellular antioxidant activity decrease [117].

It has been exposed that numerous plant extracts, due to their phytochemical compounds, have antioxidant action and roles in the antihyperglycemic activity and diabetes management [118]. Interestingly, depending on the gall-inducing species and the host plant species, it was noticed that some galls have higher carotenoid and polyphenol concentrations, which might be mechanisms to maintain oxidative homeostasis [119]. The oak galls manifested promising in vivo results against diabetic complications in thyroid gland functions [31]. The treatment with a *Q. infectoria* gall extract (500 mg and 1000 mg/kg bw for 15 days) significantly ameliorated the concentrations of both thyroid hormones, triiodothyronine (T3) and thyroxine (T4), demonstrating positive outcomes in the function of the thyroid gland usually impaired in diabetes. Also, the treatment induced an antihyperglycemic effect in diabetic rats, significantly decreasing serum blood glucose almost to normal levels. Lower intestinal glucose absorption or increased insulin secretion could be the mechanisms involved in this action [120].

Furthermore, oak galls showed wound healing beneficial effects in diabetic animals [30]. The administration of an ointment prepared from a hydroethanolic extract of *Q. infectoria* galls activated open wound healing in a diabetic mouse model by increasing collagen deposition, antioxidant capacity, and cellular proliferation, while the concentrations of malondialdehyde and proinflammatory IL-6 and TNF-α cytokines were decreased. A past study also revealed that a pharmaceutically formulated topical agent based on the antibacterial and antioxidant activities of a *Q. infectoria* gall extract enhanced the wound healing process in diabetic rats [121].

Non-steroidal anti-inflammatory drugs (NSAIDs) are commonly used to treat pain and fever via inhibiting the activity of cyclooxygenase enzymes (COX-1 and COX-2). Paracetamol (acetaminophen) is an NSAID that, for an acute overdose, could cause hepatotoxicity manifested with hepatic glutathione depletion, significant oxidative stress, and inflammatory effects [122]. Recent findings revealed that various plant extracts reduced paracetamol-induced toxicity through hepatoprotective and antioxidant activity mechanisms [123]. Likewise, oak galls showed promising results. Thus, the treatment with a *Q. infectoria* gall extract, 250 mg/kg/day for 3 consecutive days, significantly defended against paracetamol-induced toxicity via reducing oxidative stress and inflammatory and tissue-damaging effects (*p* < 0.001) in mice [4]. Moreover, the same doses of an oak gall extract lowered serum cholesterol and triglycerides, and restored serum albumin, denoting cellular preventive and tissue-protective effects [23]. Additionally, hyperlipidemic rabbits fed *Q. infectoria* gall extracts had significantly (*p* < 0.001) decreased plasma levels of TC, LDL, and TG, revealing the atherogenic and hypolipidemic activities of oak galls [124].

These findings are in agreement with recent studies reporting that natural flavonoids, such as naringenin and kaempferol, or flavonoid glycosides, identified and quantified in galls of *Quercus* species (Table 2), presented hepatoprotective effects in several animal species due to the antioxidant, anti-inflammatory, and anti-apoptotic activities [125,126].

Carcinogenic substances associated with environmental pollution could lead to uncontrolled growth of cutaneous cells into squamous cell carcinoma (SCC), a common type of epidermal neoplasia [127]. A new study disclosed that in mice orally treated with 2 g/kg of a *Q. infectoria* gall extract, the mouse skin induced tumorigenesis showed a significant reduction in tumor incidence and yield, as well as the number of papilloma, besides a significant increase in the average latent period as compared to the control group [29]. The antitumor activity showed with the *Q. infectoria* gall extract could be due to its phytochemical profile. Hence, roburic acid, a tetracyclic triterpene acid isolated from oak galls, exhibited anti-inflammatory activity and antitumor effects through inhibition of the TNF-induced NF-κB signaling pathway. Moreover, it displayed antitumor activity both in vitro and in vivo by stimulating G0/G1 cell cycle arrest and apoptosis in colorectal cancer cells [128].

Tannic acid, another bioactive compound found in oak galls in the form of gallotannins as previously discussed, was demonstrated to exert antioxidant and anti-inflammatory activity via its many hydroxyl groups, as well as anticancer action by inducing apoptosis in several cancer cell types [129]. Moreover, the cumulative concentrations of polyphenols from crude extracts of *Q. floribunda* galls in various solvents exhibited in vivo anti-inflammatory, analgesic, and antipyretic activities, as well as in vitro antioxidant capacity [130].

A recent study performed on human skin showed that an emulsion enriched with a *Q. infectoria* gall extract had potent antioxidant capacity and improved the mechanical properties of skin, including moisture and elasticity improvement, and reduced pores and sebum levels, which could have anti-aging effects [12]. In addition, the topical administration of a gall-extract-enriched emulsion possessed an anti-inflammatory effect and wound healing activity due to the capacity to modify the energy metabolism and protein production in bacteria, such as *S. aureus* [131]. These findings are in line with the results of Kaur et al. [132], which indicated in vivo anti-inflammatory activity of *Q. infectoria* gall extracts; the topical application controlled ear inflammation, while oral extract administration significantly suppressed carrageenan, histamine, and prostaglandin E2 induced paw edemas.

ROS and the inflammatory process associated with venous injury affecting the valves and venous wall induce senescence in various cell populations, including keratinocytes and fibroblasts, impeding wound healing in patients with varicose ulcers [133]. Through the excellent antimicrobial, antioxidant, and anti-inflammatory properties and through the ability to promote wound healing [24], *Q. infectoria* gall extracts could be included in preparations intended for the topical treatment of varicose ulcers, a pathology often encountered in the elderly with peripheral venous circulation problems. The wound-healing potential of topical treatment with *Q. infectoria* galls has also been demonstrated previously in an animal model of a diabetic foot [30,121].

It is encouraging that the aqueous extracts of oak galls did not induce lethality and acute toxic effects in mice (the maximum tolerance dose > 10 g/kg bw for rectal administration). Also, the extracts did not induce local mucosal irritation at the level of the colon and anal tissues in rabbits in the doses tested (the rabbit being the most sensitive species for this test) and there was no chronic toxicity or mortality in the groups of exposed Wistar rats compared to the control group [134].

However, due to the fact that higher doses (500 and 1000 mg/kg bw) of oak gall extracts were found to cause microscopic lesions in some rat tissues, including the liver, kidneys, heart, or lungs, after daily repeated exposure (28 days), the maximum dosage level should be limited [1].

## 4. Conclusions

In the last decades, plant-derived extracts have received increased attention, the existing scientific evidence underlining their important contribution in the prevention and/or treatment of various diseases, many of these diseases having oxidative stress as the basis of their etiology.

In this sense, the present systematic review summarized the literature available from the last 5 years that reported data on the phytochemical profile and pharmacological effects of the extracts obtained from the galls of *Quercus* sp. This literature review resulted in a comprehensive report on the phytochemical composition of this plant matrix and its health benefits, with a view of exploiting it as an important natural source in phytotherapy and pharmacotherapy. As highlighted in our analysis, the biological properties of oak galls can be attributed to their diverse and rich phytochemical profile, as the predominant representatives are phenolic acids, flavonoids, and hydrolyzable tannins, followed by other phenolic constituents, i.e., hydroxyphenols, coumarins, phenolic aldehydes, naphthodianthrones, acyl-phloroglucinols, and phenolic alcohols, but also non-phenolic constituents, i.e., terpenes and terpenoids, lipid compounds, carboxylic acids, and minerals. In vitro experiments have highlighted the strong antioxidant capacity and anti-inflammatory effects and antibacterial, antifungal, antimalarial, and antitumor activities of oak gall extracts, as well as the anti-aging skin properties. Promising results have been obtained in the last 5 years with metal nanoparticles (silver, zinc, copper) prepared with green methods using oak gall extracts, particularly antibacterial efficacy, increased efficacy in wound healing, as well as anticancer and anti-aging potential. In vivo experiments confirmed the outcomes obtained with in vitro studies, such as antioxidant and anti-inflammatory capacity or the anticarcinogenic activity, and also revealed the hypoglycemic potential. Furthermore, oak gall extracts exposed in vivo antibacterial activity with wound healing and skin protection effects and even improved the retention of gum tissue in human patients diagnosed with gingivitis by inhibiting the growth of oral bacteria and by having an anti-inflammatory effect. Notably, the aqueous extract of *Q. infectoria* galls did not show evident toxicity signs and mortality in acute and chronic treatment in rodents.

Further pharmaceutical, in vivo, and clinical scientific investigations are needed to incorporate the oak gall extracts into proper pharmaceutical forms and exploit them for therapeutic or cosmetic purposes. Based on the present systematic review, oak galls can be considered a likely candidate for the management of various pathologies, mainly associated with oxidative stress and chronic inflammation. Future human research should confirm the preclinical evidence and find causality between bioactive compounds from *Quercus* galls and prevention or eventual treatment of some age-related diseases including cardiovascular pathologies, type 2 diabetes, or cancer.

## Figures and Tables

**Table 1:** Characteristics of the selected studies.

Ref.	Country, Year of Publication	Study Type	Study Purpose	Plant Materials/Extraction Procedures/Formulations	Analysis Methods/Biological Systems/Animal Models/Participants	Study Outcomes/Biological Activities
[4]	Egypt, 2023	In vivo	Antioxidant, anti-inflammatory, and tissue-protective effects of Aleppo oak gall (AOG) extract against paracetamol-toxicity-induced oxidative tissue damage in mice	AOG—macerated in hydro-alcohol (80%) at room temperature for 48 h	Acetaminophen-induced hepatotoxicity experimental model in white albino mice: negative control, positive control (paracetamol—250 mg/kg/day, i.p., 3 days), and treated group (paracetamol—250 mg/kg/day, i.p., 3 days, and AOG extract—250 mg/kg/day, oral, 3 days) Biomarkers analyzed: MDA, TAC, CAT, LDH, and IL-6 in the serum of mice	AOG treatment—significant protection against - acetaminophen-toxicity-induced oxidative stress effects (*p* < 0.001 for both MDA, TAC, and CAT) - inflammatory effects (*p* < 0.001 for IL-6) - tissue-damaging effects (*p* < 0.001 for LDH), with the normalization of the values of all biomarkers analyzed (vs. control)
[23]	Saudi Arabia, 2023	In vivo	Protective effects of AOG extract against paracetamol-induced hepatotoxicity and tissue damage in mice	AOG—macerated in hydro-alcohol (80%) at room temperature for 48 h	Paracetamol-induced hepatotoxicity experimental model in white albino mice: negative control, positive control (paracetamol—250 mg/kg/day, i.p., 4 days), and treated group (paracetamol—250 mg/kg/day, i.p., 3 days, and AOG extract—250 mg/kg/day, oral, 3 days) Liver function assays: ALT, AST, and albumin in serum Assay of serum lipids: TC and TG Liver histological analysis	AOG extract—significant protective effects against acute paracetamol toxicity and restoring the serum levels of the analyzed parameters near normal: - ↓ ALT (*p* < 0.001) and ↓ AST (*p* < 0.001) to normal state - ↑ albumin (*p* < 0.001); restored it to baseline - ↓ TC (*p* < 0.001) and ↓ TG (*p* < 0.001) to normal state AOG extract—liver protection against the damaging effects induced with paracetamol toxicity: minimal residual degenerative changes and the absence of necrosis
[12]	Pakistan, 2023	In vitro In vivo (humans)	Antioxidant effects, effects on skin mechanical properties, and anti-aging effects of *Quercus infectoria* (QI) (Olivier) emulsion	QI galls—macerated in methanol, ethanol, acetone, and distilled water for 7 days Formulations: - QI-loaded emulsion: QI extract (4%) combined with 10% stearic acid, 2% acetyl alcohol, and 14% glycerin - Control emulsion	Quantitative determination of total secondary metabolites of QIGE: TPC and TFC Antioxidant activity (AA) assay of the QIGE: DPPH assay Tyrosinase enzyme inhibition activity; sun protection factor Organoleptic evaluation, spread ability, pH, conductivity measurements, and rheological studies of test and control formulations Noninvasive in vivo study—13 participants (women, aged 22–35), for 12 weeks: patch test; panel test; evaluation of skin mechanical properties	QIGE: - TPC: 56.1 mg GAE/g; TFC: 35.32 mg QE/g; tyrosinase enzyme inhibition activity of 76% - AA: 81% vs. reference (ascorbic acid) - sun protection factor of 19 QI-extract-enriched emulsion: - no adverse side effects or hypersensitivity - an average reduction of 40% in the small pore count and 73% in the big pore count - ↓ sebum level vs. control emulsion (*p* = 0.01) - ↑ moisture level of the skin by 85% vs. control emulsion (*p* = 0.01 at 12 weeks) - considerable improvement in skin elasticity, the elasticity level increasing by 12% vs. control emulsion
[24]	Iran, 2023	In vitro In vivo	Antimicrobial, antioxidant, and wound healing properties of electrospun nanofiber CuNPs and QI gall extracts (QIGE)	QI galls—macerated in methanol (80%) in a ratio of 1:6 (*w*/*v*) for 48 h CuNPs—synthetized using *Calendula officinalis* flowers’ extract	Characterization of the biosynthesized CuNPs: DLS, FT-IR, XRD, and FESEM techniques Characterization of the nanofibers: FT-IR and FESEM techniques Quantitative determination of total secondary metabolites of QI galls’ extract: TPC and TFC Antibacterial activities of the biosynthesized CuNPs and QI galls’ extract: resazurin viability assay Testing wound dressing characteristics: mechanical strength, water vapor transmission rate, swelling ability, and degradation rate AA assay of the nanofibers: DPPH assay Antibacterial activity of the nanofibers against MRSA: resazurin viability assay Cytotoxicity of the prepared nanofibers against human dermal fibroblast cells: MTT assay In vivo studies on wounds experimentally induced and infected with MRSA (adult Wistar rats): wound healing performances; antibacterial activity; histological analysis	FESEM: the nanofibrous structure with the average diameter of 152.81 nm for PCL/PVA/QI galls/CuNPs PCL/PVA incorporated with CuNPs (6 × MIC) and QI galls (4 × MIC)—the most remarkable antibacterial, antioxidant, and cellular biocompatibility performances PCL/PVA/QI galls/CuNPs: - high potential to protect the wound area from possible infection, absorbing wound exudates and facilitating gas exchange - 77.6 and 73.8% wound healing in non-infected and MRSA-infected wounds, respectively, on the 5th day of the wound closure assay - complete skin regeneration healing in treated wounds and less inflammation, confirmed with histological assessment, on the 10th and 15th days
[11]	Iran, 2023	In vitro	Antimicrobial activity of QIGE against cariogenic microorganisms	QI galls—extracted in ethanol/water (80:20, *v*/*v*) at room temperature for 24	Antimicrobial activity of QIGE compared to *Scrophularia striata* extract against *Streptococcus mutans* (ATCC 35668), *S. sobrinus* (ATCC 27607), and *C. albicans* (ATCC 10231): resazurin colorimetric assay MIC of the QI galls’ extract MBC of the QI galls’ extract	QIGE—efficient antimicrobial activity: - more potent in inhibiting growth and killing the microorganisms, compared to the *S. striata* extract - the MIC values against microbial species in the range of 0.039–0.625 mg/mL - *S. sobrinus*—the most susceptible - *C. albicans*—the least inhibited by the extract - MBC > MCI for all; in the range of 0.312–5 mg/mL
[25]	Iraq, 2023	In vitro	Antifungal activity of the alcohol QIGE against *Penicillium expansum* and *Aspergillus flavus*	QI galls—extracted with 2% acetic acid in a ratio of 1:4 at 70 °C for 8 h; phenolic compounds separated with n-propanol; final concentration of QI gall samples: 100, 200, and 300 mg/mL	Antifungal activity of phenolic compounds’ extracts: the mixing method with Sabouraud dextrose agar Determination of the percentage inhibition of diameter growth (PIDG)	All extracts—inhibitory activity against *P. expanisum* and *A. flavus* Increase in PIDG—with increasing concentrations: - on *P. expansum*—PIDG: 16.78% for all concentrations; PIDG = 31.1 ± 3.335% at 300 mg/mL - on *A. flavus*—PIDG: 51.48%; PIDG = 62.49 ± 3.63% at 300 mg/mL
[10]	Turkey, 2023	Phytochemistry In vitro	Phenolic composition of the gall extracts of *Andricus sternlichti* Bellido. Antioxidant, cytotoxic, and anti-apoptotic effects of extracts of *A. sternlichti* galls	*A. sternlichti* galls—extracted in organic solvents (acetone, ethanol, methanol, and distilled water) at 55 °C for 6 h	Quantitative determination of total secondary metabolites of galls of *A. sternlichti*: TPC, TFC, and TCT Identification and quantification of phenolic compounds using HPLC-DAD Total AA: β-carotene-linoleic acid assay; phosphomolybdenum assay. Radical scavenging activity: DPPH, ABTS. Reducing power activity: CUPRAC, FRAP, metal chelating activity Cytotoxic activity on the MIA PaCa-2 cell lines Anti-apoptotic activity: Bax, Bcl-2, FAS, Bid, Caspase-3, Caspase-8, Caspase-9, Caspase-10, FADD, TRADD gene expression using RT-PCR	The methanol extract—the highest TPC (319.97 ± 7.29 mg GAE/g); the water extract—the highest TFC (11.86 ± 0.66 mg QE/g); the acetone extract—the highest TCT (43.75 ± 1.81 mg CE/g) A total of 15 phenolic compounds identified and quantified in methanolic gall extracts: 11 phenolic acids and 4 flavonoids The most abundant components: caffeic acid (589.042 mg/g), ellagic acid (261.998 mg/g), and epicatechin (171.498 mg/g) AA: ethanol extracts—the highest total antioxidant and reducing power activity; the methanol extract—the strongest radical scavenging activity Antiproliferative activity via regulating expressions of apoptotic genes: ethanol extract—with antiproliferative effect on MIA Paca2 cell lines at low concentrations
[9]	Indonesia, 2023	Phytochemistry In vitro	The effects of extraction with supercritical CO_2_ and methanol co-solvent on phenolic composition and toxicity of QIGE	CSE: QI galls—extracted in methanol (1:10, *w*/*v*) at 50 °C for 8 h SCFE-CO_2_: QI galls—extraction into CO_2_/methanol (500:1), at 20 MPa for 60 min, flow rate of 25 mL/min	Quantitative determination of polyphenols: TPC Identification and quantification of phytochemicals using LC-MS/MS Cytotoxicity of QIGE on Vero cells: MTT assay	Composition of QIGE: - qualitative LC–MS analysis: 12 peaks in the SCFE-CO_2_ extraction without co-solvent vs. 27 peaks with co-solvent TPC: 1596 ± 41 mg GAE/100 g after CSE vs. 1799 ± 13 mg GAE/100 g after SCFE-CO_2_ extraction with the highest proportion of co-solvent Cytotoxicity: the highest for CSE extract (IC_50_: 713 ± 86 μg/mL) vs. SCFE-CO_2_ extracts obtained with co-solvent (IC_50_ in the range of 2685 ± 51 and 1335 ± 82 μg/mL) or without (2945 ± 92 μg/mL)
[8]	India, 2023	Phytochemistry In vitro	Phytochemical analysis of active constituents in QIGE. Antibacterial effects of topical formulations containing QIGE and AgNPs against Gram-positive and Gram-negative bacteria	QI galls—extraction in 50% (*v*/*v*) ethanol, in a ratio of 1:5 (*w*/*v*), at 70 °C for 10 h AgNPs—synthesized using QI gall extract	Qualitative analysis of the constituents: carbohydrates, amino acids, proteins, saponins, alkaloids, glycosides, flavonoids, phenolic compounds, and tannins Identification and quantification of active constituents in QIGE using HPLC-DAD Antibacterial activity: agar well-diffusion method against *Staphylococcus aureus*, *Pseudomonas aeruginosa*, and *Escherichia coli* MIC: serial microdilution broth assay	QIGE content: tannins, carbohydrates, amino acids, and proteins, a large amount of tannic acid and smaller amounts of gallic and ellagic acids Significant activity against Gram-positive and Gram-negative bacteria: - *S. aureus*, *P. aeruginosa*, and *E. coli* (zone of inhibition of 30 mm, 26 mm, and 24 mm, respectively, compared with streptomycin, 22 mm) - better antibacterial activity for a concentration of 10 mg/mL (chosen for formulation) AgNPs: higher antibacterial activity vs. gall extract The formulated gel: - drug delivery for more than 4 h - not irritating - higher antibacterial activity vs. the marketed gel formulation containing tannic acid for the treatment of mouth ulcers and gingival disorders
[14]	Saudi Arabia, 2022	Phytochemistry In vitro	Identification of the phytochemical constituents of QIGE. Antibacterial activity of QIGE and its nano-form on *Helicobacter pylori*	QI galls—extracted with ethanol in a ratio of 1:10 (*w*/*v*) QI-ZnO-NPs—synthesized using QIGE	Identification of QIGE constituents, hydrolysable tannins, gallic acid dimers, gallic acid trimers, phenolic acids, using LC-MS/MS Characterization of Qi-ZnO-NPs using UV, IR, DLS, TEM, and SEM measurements Antibacterial activity against *H. pylori* (ATCC-43526 strain) vs. amoxicillin and clarithromycin: agar diffusion method	A total of 20 compounds identified—as major gallic acid conjugates Activity against *H. pylori*: - moderate activity for both QIGE and Qi-ZnO-NPs - higher inhibition for Qi-ZnO-NPs (98.4%) vs. amoxicillin (93.2%) and clarithromycin (90.7%) - amoxicillin:QI-ZnO-NPs (1:2 and 1:4): synergism with ↓ MIC_90_ of two-fold and four-fold, respectively
[26]	Iran, 2022	In vitro	Antimicrobial effects of the QIGE and its copper oxide nanoparticles against Gram-positive and Gram-negative bacteria species	QI galls—extracted with water in a ratio of 1:5 for 5 min at 85 °C CuO-NPs—synthesized using aqueous QIGE	Characterization of CuO-NPs: FT-IR, XRD, DLS, SEM, EDAX, TEM, and TGA techniques Antibacterial activity against *Bacillus cereus* (ATCC 14579), *S. aureus* (ATCC 12600), *P. aeruginosa* (ATCC 10145), *E. coli* (ATCC 11175), *Acinetobacter baumannii* (ATCC 19606), and *Klebsiella pneumonia* (ATCC 13883), vs. chloramphenicol and penicillin: agar well-diffusion method MIC: macrobroth dilution method	CuO-NPs—average size of 20 nm Antibacterial activity: - very high activity for both QIGE and CuO-NPs against all tested bacteria species (CuO-NPs > QIGE) - the highest antibacterial activity against *K. pneumonia* - the lowest antibacterial activity against *P. aeruginosa* - the MIC values of CuO-NPs—lower than those of the extract
[27]	Iran, 2022	In vitro	Antimicrobial, antioxidant, and anticancer properties of the aqueous QIGE and its silver nanoparticles	QI galls—extracted with water in a ratio of 1:4 for 5 min at 90 °C AgNPs—synthesized using QIGE	Characterization of AgNPs: UV–Vis spectrophotometry, FT-IR, TEM, DLS, XRD, and TGA techniques Antibacterial activity against *Enterococcus faecalis*, *S. aureus*, *P. aeruginosa*, and *K. pneumonia*: agar well-diffusion method MBC: standard broth dilution method AA: DPPH assay Cytotoxic activity against human breast adenocarcinoma (MCF-7) cells: MTT assay	AgNPs—an average diameter of 33 nm Antibacterial activity: - high antibacterial potential for both QIGE and AgNPs against all tested bacteria species (AgNPs > QIGE) - the highest antibacterial activity against *K. pneumonia* - the lowest antibacterial activity against *P. aeruginosa* and *S. aureus* - the MBC values of AgNP—lower than those of QIGE Antioxidant activity: AgNPs > QIGE (IC_50_: 109.13 ± 0.52 μg/mL vs. 264.00 ± 0.02 μg/mL) Anticancer activity against MCF-7 cells: - strong cytotoxic activity of AgNPs: cellular viability of 16.12, 11.78, and 14.77% at different concentrations of AgNPs (125, 375, and 625 μg/mL, respectively)
[1]	Iran, 2022	In vivo	Acute and repeated oral toxicity of the hydroalcoholic extract of *Q. brantii* galls in rats	*Q. brantii* galls—extracted in ethanol/water (70:30) in a 1:20 ratio for 24 h at room temperature	Female Wistar rats treated with *Q. brantii* gall extract: - acute toxicity—with gavage: 2000 mg/kg bw - repeated oral dose toxicity—with gavage: 50, 500, and 1000 mg/kg bw/day, for 28 days	Oral acute toxicity: LD_50_ > 2000 mg/kg bw Repeated oral dose toxicity test: - no evident toxicity and mortality at any of the doses tested - small changes in some biochemical (TSH, T3, and T4) and hematological parameters (MCHC and MCH) observed in the rats treated with 500 or 1000 mg/kg bw/day - slight tissue damage (in the liver, kidneys, stomach, heart, spleen, lungs, uterus, and ovary) in rats treated with 500 or 1000 mg/kg bw/day
[28]	Malaysia, 2021	In vitro	Cytotoxic effects and the cell death mechanisms of aqueous QI and SCFE-CO_2_ on HeLa cervical cancer cells	Aqueous extraction: QI galls—extracted with water in a ratio of 1:4 for 24 h at 50 °C SCFE-CO_2_: QI galls—extracted at 50.4 °C and 2508 PSI	Cytotoxicity: MTT assay Apoptosis induction: acridine orange/propidium iodide staining Phosphatidylserine externalization: Annexin V-FITC Apoptosis Detection Kit 1 Cell cycle distribution: CycleTEST^TM^ PLUS DNA Reagent Kit Caspase activity: FAM FLICA^TM^ Caspases Kit Expression of p53, Bax, and Bcl-2: anti-Bax, anti-Bcl-2, and anti-p53 antibody FITC and Cell Fixation and Permeabilization Kit	Aqueous and SCFE-CO_2_ QIGE—cytotoxic effects towards HeLa cell line: IC_50_ of 12.33 ± 0.35 μg/mL and 14.33 ± 0.67 μg/mL, respectively In the cells treated with both extracts: - observed morphological changes - apoptosis activation with activation of caspase-8 and caspase-9, enhancing the expression of pro-apoptotic p53 and Bax, inhibiting the expression of anti-apoptotic Bcl-2 - cell cycle progression arrest with SCFE-CO_2_: better than aqueous QIGE through the activation of cell cycle arrest at sub G0 phase
[29]	Iraq, 2021	In vivo	Antitumor activity of the aqueous QIEG on DMBA-induced mouse skin tumorigenesis	QI galls—extracted in water for 24 h at 45 °C	DMBA/croton-oil-induced skin carcinogenesis experimental model in mice (male Swiss albino mice, strain Balb/c): QIGE (orally, 2 g/kg bw/day) was administered as pre-treatment (alone) for 7 days before DMBA application (0.1% in acetone, locally, in a single dose) and/or as post-treatment—3×/week for 16 weeks, 14 days after the DMBA application, in association with croton oil (1 mL/100 mL acetone, locally) Measurement of body weight: daily Morphological and histopathological examination of tumors	Significant antitumor activity of QIGE: - ↓ tumor incidence, tumor burden, tumor yield, and cumulative number of papilloma - ↑ average latent period in mice treated with QIGE - ↓ histopathological alterations: epidermal hyperplasia, keratinized pearl formation, and acanthosis in skin and tumors
[30]	Iran, 2021	In vivo	The effects of topical application of hydroethanolic QIGE on open wound healing in a streptozocin-induced diabetic mouse model	QI galls—extracted with hydroethanolic solution in a ratio of 1:4 for 96 h Ointments—5% and 10% QIGE, respectively	Streptozocin-induced diabetic BALB/c mice (55 mg/kg bw, for 4 days); two circular wounds (5 mm) on the dorsum of the mice Histopathological and IHC analysis Biomarkers of OS: TAC, TTM, and MDA in the wound tissue. Biomarkers of inflammatory condition: TNF-α and IL-6 in the serum of mice (Elisa kit) Molecular analysis: mRNA levels of VEGF, p53, and Bcl-2—RT-PCR	Ointments—acceleration of healing of open wound with - AA: ↑ tissue TAC and TTM levels, and ↓ MDA level (*p* < 0.05) - ↓ immune cell infiltration, and TNF-α and IL-6 levels (*p* < 0.05) - inducing apoptosis: ↑ m-RNA level of p53 (*p* < 0.05) - up-regulating cellular proliferation: ↑ Bcl-2 expression in connective tissue cells (especially fibroblasts, fibrocytes, and endothelial cells) (*p* < 0.05) - up-regulating angiogenesis: ↑ mRNA levels of VEGF, p53, and Bcl-2 in a dose-dependent manner (higher for 10% QIGE than for 5%)
[31]	Iraq, 2021	In vivo	The effect of QIGE on the thyroid gland and testicular functions in diabetic rats	QI galls—macerated with 80% methanol at 25 °C for 2–3 days	Streptozocin-induced diabetic rat model (55 mg/kg bw, single dose): QIGE administered with gavage, 500 and 1000 mg/kg bw, for 15 days Biochemical parameters: serum blood glucose, TSH, T3, T4, T, and LH Histopathological analysis: thyroid gland and testis IHC analysis: expression of TTF-1 in the thyroid gland of rats (the TTF-1 monoclonal antibody kit)	QIGE (both 500 and 1000 mg/kg bw): - antihyperglycemic effect - ↑ T3 and T4 - no effect on testicular function - almost completely restoring the morphological alterations to normal in the thyroid gland and testis - restoring the overexpression of TTF-1 to normal in the thyroid gland
[32]	Malaysia, 2021	In vitro	Cytotoxicity- and apoptosis-inducing activity of ethyl acetate QIGE in HeLa cells	QI galls—extracted with ethyl acetate in a ratio of 1:5 for 72 h	Cytotoxicity: MTT assay Apoptosis induction: acridine orange/propidium iodide staining Phosphatidylserine externalization: Annexin V-FITC Apoptosis Detection Kit 1 Cell cycle distribution: CycleTESTTM PLUS DNA Reagent Kit	Ethyl acetate QIGE: - cytotoxicity effect (IC_50_ of 11.50 ± 0.50 μg/mL) - induction of apoptosis (from 1.00% to 10.33%) - early apoptosis observed in annexin V/propidium iodide staining - apoptosis confirmed with an increase in cell population in sub G0/G1 phase
[15]	Malaysia, 2021	Phytochemistry In vitro	Identification and quantification of gallotannin in crude and fractionated QIGE. Antioxidant and cytotoxic effects of a gallotannin-enriched fraction from QI galls on GBM	QI galls—extracted with water at ~100 °C for 6 h Six fractions (F1–F6) in methanol (0%, 10%, 25%, 50%, 75, and 100%) obtained from aqueous QIGE	Gallotannin—TLC and HPLC-DAD AA: DPPH assay; reducing power assay Cytotoxicity (MTT assay) on the human GBM cells (DBTRG-05MG) of QIGE and its F4 fraction.	Antioxidant activity: - F4 fraction: ↑ AA in both assays vs. reference synthetic pure compounds (*p* < 0.05) Cytotoxicity: - F4: a similar inhibitory effect on GBM with Temozolomide and Tamoxifen (IC_50_ of 15.0 μg/mL vs. 13.9 μg/mL and 14.0 μg/mL, respectively)
[33]	Malaysia, 2021	Phytochemistry	The effects of extraction solvents on the overall phytochemical content, recovery of tannin, and AA of QIGE	Methanol extraction: QI galls—extraction with methanol in a ratio of 1:10 at ~64 °C for 6 h Aqueous extraction: QI galls—extraction with water in a ratio of 1:10 at ~100 °C for 6 h	Qualitative analysis of the constituents: phenolic compounds, tannins, hydrolysable tannin, non-hydrolysable tannin, alkaloids, flavonoids, saponins, terpenoids, quinines, triterpenes, cardiac glycosides Gallotannin—TLC and HPLC-PDA AA assay: DPPH	Qualitative analysis: - ↑ yield of crude extract powder with methanol vs. water as solvent - methanol extract—richer in flavonoids - aqueous extract—richer in phenolic compounds, tannins, gallotannin, triterpenes, and cardiac glycosides - QIGE content: only gallotannin (hydrolysable tannin) - water: optimal solvent for extracting the tannin compound—75.0 μg/mL vs.−46.8 μg/mL for extraction in methanol - higher AA for aqueous vs. methanolic extract
[34]	Iran, 2021	Phytochemistry In vitro	Phytochemical screening and quantification of constituents in the hydroalcoholic QIGE. Antioxidant and antibacterial activities	QI galls—macerated with ethanol/water solvent (70/30) for 72 h	Qualitative analysis of the constituents: phytochemical tests Quantitative determination of TPC, TFC Identification and quantification of phenolic compounds in the extract: HPLC-PDA AA assay: DPPH Antibacterial activity against *Bacillus pumilus* (PTCC 1274), *B. subtilis* (ATCC 9372), *S. aureus* (ATCC 25923), *B. cereus* (PTCC 1015), *K. pneumoniae* (ATCC 3583), *E. faecalis* (ATCC 15753), *E. coli* (ATCC 25922), *S. epidermidis* (ATCC 12228), *P. aeruginosa* (ATCC 27852) compared to tetracycline, ampicillin, and gentamicin: disk diffusion method MIC: microdilution assay	The hydroalcoholic QIGE: - presence of alkaloids, flavonoids, tannins, saponins, and phenolic compounds (3 phenolic acids: gallic, benzoic, and caffeic acids; 2 flavonoids: rutin and quercetin) - TPC of 16.21 mg/g and TFC of 1.78 mg/g dried galls - AA: IC_50_ of 47 μg/mL - high activity against *E. coli*, *K. pneumonia*, *S. aureus*, and *S. epidermidis*, with higher MIC than tetracycline
[35]	Malaysia, 2021	In vitro	Antimalarial effect of the acetone crude QIGE	QI galls—extracted with acetone, methanol, ethanol, or water in a ratio of 1:5, for 72 h at 50 °C	Quantitative measurement of the pH of the malaria parasite digestive vacuole: flow cytometry	A novel mechanism of action of QIGE against *Plasmodium falciparum*: - significant increase in the pH of the digestive vacuole for the acetone extract (with 1.03, 1.23, and 1.39 pH units) in a concentration-dependent manner (at 35.1, 70.2, and 140.4 μg/mL)
[36]	Malaysia, 2021	In vitro	Immunomodulatory potential of the water QIGE	QI galls—macerated in water in a ratio of 1:5 for 72 h at 50 °C; final concentrations of 16, 32, and 64 μg/mL	Proliferative activity of the QIGE on the murine macrophage (J774A.1) cell line: MTT assay Phagocytic activity of extract-treated macrophages: flow cytometry NO production with extract-treated macrophages: Griess reaction The levels of pro- and anti-inflammatory cytokines in the macrophage culture: enzyme-linked immunosorbent assay	QIGE: - non-toxic on J774A.1 cell line - ↑ proliferation (maximum of 154.2 ± 0.1% at 64 μg/mL after 72 h) and phagocytosis (from 55.1% (untreated cells) to 74.2% (macrophages treated with 64 μg/mL extract)) of macrophages - ↓ NO production in a dose-dependent manner (maximum at 64 μg/mL) - regulation of the cytokine levels in macrophages: ↓ iNOS and NO levels, as well as IL-4, IL-6, and IL-12 cytokines, and ↑ IL-13 and other cytokines (IL-2, TNF-α, IL-5, IL-10, IL-23, TGF-β1, and IL-17A)
[37]	Iraq, 2020	In vivo (humans)	The effects of QI galls as oral powder in patients with both gingivitis and dental plaque	QI galls—powder	Ten participants (nine women and one man, aged 25–55) The diagnoses and follow-up treatment: in a dental clinic under the supervision of a specialist dentist, according to the standards of Plaque Index and Gingival Index	QIGE: - ↓ the progress of plaque after 2 weeks of treatment, measured in terms of mean, population SD, and variance to 0.29, 0.12, 0.01 vs. 0.66, 0.17, 0.02, respectively, before treatment - ↓ the progress of gingivitis after 2 weeks of treatment to 0.32, 0.11, 0.01 vs. 0.72, 0.15, 0.02, respectively, for mean, population SD, and variance
[38]	Iraq, 2020	In vivo	Anticlastogenic effect of QIGE against DMBA-induced genotoxicity in bone marrow cells of mice	QI galls—extracted in water in a ratio of 1:4 for 24 h at 45 °C	DMBA-induced genotoxicity experimental model in mice (male Swiss albino mice, strain Balb/c): DMBA (50 mg/kg bw, i.p., single dose) Acute toxicity of QIGE—gavage: 2, 4, 6, 8, 10, and 12 g/kg bw Cytogenetic biomarkers: mitotic index, chromosome aberration, and micronuclei	QIGE: - no signs of toxicity even at 12 g/kg bw; LD_50_ could not be calculated - strong anticlastogenic effect: ↓ the number of bone marrow micronuclei induced with DMBA; ↓ the number of metaphases with chromosomal aberrations; ↑ the mitotic index vs. positive control group
[39]	India, 2020	In vitro	Anticariogenic activity of the galls of QI (Olivier) against oral pathogens causing dental caries	QI galls—extraction in water, methanol, ethanol, ethyl acetate, acetone, or hexane in a ratio of 1:5, for 6 h	Antimicrobial activity of the extracts against *C. albicans* MTCC 183, *S. mutans* MTCC 497, *Lactobacillus acidophilus* MTCC 10307, and *S. aureus* MTCC 1144: agar well-diffusion method MIC: two-fold serial microdilution method Inhibition of streptococcal biofilm: shell assay; microtiter plate assay	All QIGE extracts—antimicrobial activity (methanol > ethanol > acetone > other solvents used): - MIC values of the methanolic extract against each bacterial species: in the range of 0.16 to 0.31 mg/mL - *S. aureus*—the most susceptible bacteria (with the lowest MIC value), *C. albicans*—the least inhibited - ↓ the growth of streptococcal biofilm (maximum effect at 0.018 μg/mL of methanolic extract)
[40]	Turkey, 2020	Phytochemistry In vitro	Phenolic composition of the gall extracts of *Andricus tomentosus*. Antioxidant, cytotoxic, and anti-apoptotic effects of extracts of *A. tomentosus* galls.	*A. tomentosus* galls—extracted with acetone, ethanol, methanol, or water at 50 °C for 6 h	Quantitative determination: TPC, TFC, TCT Phenolic compound analysis: HPLC-DAD Total AA: β-carotene-linoleic acid assay; phosphomolybdenum assay; DPPH; ABTS; CUPRAC; FRAP Cytotoxic activity on the MIA PaCa-2 cell lines: XTT assay Anti-apoptotic activity: Bax, Bcl-2, FAS, Bid, Caspase-3, Caspase-8, Caspase-9, Caspase-10, FADD, TRADD Gene expression: RT-PCR	Phytochemical analysis: - ethanol extract—highest TPC (297.47 ± 2.52 mg GAE/g) - water extract—highest TFC (46.88 ± 0.21 mg QE/g) - acetone extract—highest TCT (48.22 ± 1.09 mg CE/g) - A total of 15 phenolic compounds identified and quantified (11 phenolic acids and 4 flavonoids); most abundant components: caffeic acid, ellagic acid, and 2,5-dihydroxy benzoic acid Highest total AA: - methanol extracts (β-carotene-linoleic acid assay: 92.58 ± 0.92%, and CUPRAC: 89.81 ± 0.96 mg TE/g) - ethanol extracts (phosphomolybdenum assay: 104.36 ± 4.95 mg AE/g, and FRAP: 184.01 ± 2.83 mg TE/g) - water extracts (DPPH: IC_50_ of 9.56 ± 1.08 μg/mL, and ABTS: IC_50_ of 18.51 ± 0.25 μg/mL) - acetone extract (chelating capacity: 40.07 ± 2.30%) - Antiproliferative activity on MIA PaCa-2 cell lines: - acetone extract: the best cytotoxic effect (IC_50_ of 124.7 μM)
[41]	Thailand, 2020	In vitro	Antibacterial activity of QIGE against diarrhea-causing bacteria	Ethanol extraction: QI galls—extracted with 95% ethanol at room temperature for 7 days Aqueous extraction: QI galls—boiled for 2 h	Antibacterial activity against food isolates of *S. aureus* (*n* = 11), *Vibrio cholerae* (*n* = 10), *V. parahaemolyticus* (*n* = 10), and against reference strains (*S. aureus* ATCC 23235, *S. aureus* ATCC 27664, and *V. parahaemolyticus* ATCC 17802), vs. penicillin and ciprofloxacin: paper disc agar diffusion method MIC: broth microdilution method	Ethanol and water QIGE: - antibacterial efficacy against all bacterial strains - the best bacteriostatic activity against *V. parahaemolyticus*, with an MIC range of 15.63–31.25 μg/mL (for ethanol extract) and 7.81–250 μg/mL (for aqueous extract)
[18]	China, 2020	Phytochemistry	Profiling and identifying chemical compounds of Turkish galls	Turkish galls—extracted with water reflux at 100 °C for 1 h	Analysis of tannins: HPLC-ESI-MS/MS	Twelve compounds identified or partially characterized: - phenolic acids (gallic acid, digallic acid, and ellagic acid) - ellagic acid derivatives (galloyl-HHDP-glucose and pedunculagin) - gallotannins (monogalloyl-glucoside, digalloyl-glucoside, trigalloyl-glucoside, tetragalloyl-glucoside, pentagalloyl-glucoside, hexagalloyl-glucoside, heptagalloyl-glucoside)
[19]	India, 2020	Phytochemistry In vitro	Phytochemical screening of QIGE and identification of antibacterial phytocompounds. Antibacterial activity against antibiotic-resistant *Salmonella Typhi* and *S. Enteritidis* of poultry origin	QI galls—extracted in ethanol, methanol, or water at the ratio of 1:20 (*w*/*v*) for 48 h	Qualitative analysis: phytochemical tests Identification of bioactive compounds: GC-MS Preliminary antibacterial activity of aqueous, ethanolic, and methanolic extract: agar disk diffusion method In vitro antibacterial screening against antimicrobial-resistant *S. Typhi* and *S. Enteritidis*: plate count method	Phytochemical analysis: - presence of tannins, cardiac glycosides, phenols, steroids, flavonoids, terpenoids, and saponins - A total of 23 phytocompounds detected using GC-MS (16 identified compounds being responsible for the antibacterial activity) Antibacterial activity: - methanolic extract > aqueous and ethanolic extracts - dose-dependent inhibition against *S. Typhi* and *S. Enteritidis*: 100% bactericidal effect (completely inhibited *S. Typhi* and *S. Enteritidis*) for the methanolic extract at 50 mg/mL and significant bacteriostatic effect at lower concentrations
[42]	Malaysia, 2020	In vitro	Antimalarial and toxicological activities of QIGE	QI galls—macerated with acetone, methanol, ethanol, or water in a ratio of 1:5 (*w*/*v*), for 72 h at 50 °C	Antimalarial activity: malarial SYBR Green I fluorescence-based assay Toxicological activity: brine shrimp lethality test; hemolytic assay Cytotoxicity (MTT assay) against normal embryo fibroblast cell line (NIH/3T3) and normal kidney epithelial cell line (Vero)	Antimalarial activity: - acetone extract (IC_50_: 5.85 ± 1.64 μg/mL) > methanol extract (IC_50_: 10.31 ± 1.90 μg/mL) > ethanol extract (IC_50_: 20.00 ± 1.57 μg/mL) > aqueous extract (IC_50_: 30.95 ± 1.27 μg/mL) Toxicity: - all the crude extracts—non-toxic on brine shrimps (LC_50_ > 1000 ppm) and on normal human erythrocytes (<5% hemolysis) Cytotoxicity: - all extracts—mildly toxic on NIH/3T3 cells and non-toxic on Vero cells
[43]	Malaysia, 2020	Phytochemistry	Performance in the extraction of phenolic compounds from oak galls using ionic liquids and their analysis	Oak galls—extractions using CAE, CUBAE, and UPAE with water, methanol, CTAB, and 2 ionic liquids: [Bmim][BF4] and [Bmim][Tf2N]; extraction time: 2–10 h	Quantification of phenolic compounds: HPLC-DAD Identification of the functional groups of the extracted bioactive compounds: FT-IR spectrometry analysis	A total of 9 phenolic compounds and 3 organic acids identified Extraction yield: - UPAE method—two times more efficient with ionic liquids: [Bmim][BF4]—481.04 mg gallic acids/g and 2287.90 mg tannic acids/g; [Bmim][Tf2N]—497.34 mg gallic acids/g and 2430.48 mg tannic acids/g, vs. without ionic liquid (130.36 mg gallic acids/g and 1556.26 mg tannic acids/g)
[44]	Malaysia, 2020	Phytochemistry In vitro	QIGE: - phytochemical screenings - cytotoxic effects on different selected human cancer cells	QI galls—successively extracted with n-hexane, ethyl acetate (QIEA), and methanol	Qualitative analysis: alkaloids, tannins, glycoside, flavonoids, terpenoids, saponins, and phenolic compounds Cytotoxicity (MTT assay) on the human cervical cancer (HeLa), breast cancer (MCF-7 and MDA-MB-231), liver cancer (Hep G2), and normal fibroblast (L929) cell lines	Cytotoxicity of QIEA (the most potent extract): - the lowest IC_50_ value against HeLa cells - cytoselective property against L929 cell line - induced apoptosis in the treated cells
[45]	China, 2020	In vitro	Antitumor activity of the aqueous QIGE on CRC cells	QI galls—extracted with water for 1 h in a ratio of 1:8 (*v*/*v*)	Cytotoxicity on the CRC human cell line (HT-29) and CRC murine cell line (CT-26): CCK8 assay Apoptosis: Annexin V-FITC/propidium iodide Apoptosis Detection Kit Autophagy: TEM, flow cytometry, laser confocal and Western blotting test The underlying mechanism of QIGE against CRC cells: Reactive Oxygen Assay kit, transwell, and wound healing tests	Cytotoxicity of QIGE: - suppressing the viability of CRC cells and triggering caspase-dependent apoptosis - triggering the autophagic cell death - induction of intracellular ROS accumulation - participation of both Erk and AKT/mTOR signaling pathways in the autophagic cell death process - influencing the epithelial mesenchymal transition process and inhibiting the migration of CRC cells
[20]	Iraq, 2019	Phytochemistry In vitro	QIGE: - identification and quantification of phytochemicals - antibacterial activity on *P. aeruginosa*	QI galls—extracted with ethyl acetate, n-butanol, ethanol, or water	Phytochemical analysis: LC-MS/MS Antibacterial activity on isolates of multiple-drug-resistant *P. aeruginosa*	Phytochemicals identified: phenolic acids, flavones, flavonols, flavanones, naphthodianthrones, and phloroglucinols Antibacterial activity of QIGE: - ↓ expression of the genes encoding quorum sensing (las, rhl) and exotoxin A, including the associated virulence and biofilm formation
[46]	India, 2019	Phytochemistry In vitro	Aqueous QIGE: - phytochemical screening - antibacterial and antibiofilm activity against *Rothia dentocariosa* isolated from dental caries	QI galls—extracted in heated water in a ratio of 1:25 (*w*/*v*) for 10–15 min	Qualitative analysis of the constituents: phytochemical tests Antibacterial activity: agar well-diffusion method Antibiofilm activity: microtiter plate assay	Phytochemical analysis: alkaloids, phenolic compounds, tannins, glycosides, and flavonoids Antibacterial activity: - aqueous QIGE—potent against *R. dentocariosa* - ↑ diameter of inhibition zone vs. chlorhexidine (19.00 ± 7.07 mm vs. 15 mm, respectively) at 100 μg/mL - the antibiofilm activity—maximum (92.89%) at 100 μg/mL
[47]	Turkey, 2019	Phytochemistry	Quantification of tannic acid in different extracts of two oak galls: QI subsp. *Boissieri* and QI subsp. *infectoria*	QI galls—extracted in a ratio of 1:20 (*w*/*v*) with (1) 96% ethanol at 45 °C for one night; (2) 80% methanol for 8 h at room temperature; (3) 70% acetone for 8 h at room temperature; (4) diethylether/ethanol/water mixture (25:3:1) for 8 h at room temperature	Quantification of tannic acid: HPLC-DAD	The content of tannic acid for QI subsp. *boissieri* and QI subsp. *infectoria* galls, respectively: - 30.852 and 81.012 mg/g (96% ethanol extract) - 43.898 and 127.683 mg/g (80% methanol extract) - 3.064 and 67.200 mg/g (70% acetone extract) - 0.016 and 0.112 mg/g (mixture of diethylether/ethanol/water)
[48]	China, 2019	In vivo	Anti-inflammatory and gut-microbiota-modulating effects of Turkish galls (TGE) in DSS-induced UC in mice	Turkish galls’ effective parts	DSS-induced experimental ulcerative colitis (UC) (Kunming mice, 4% DSS in drinking water, for 17 days): (1) normal group (NC), (2) DSS control group (DSS), (3) 5-aminosalicylic acid group (5-ASA, 50 mg/kg), (4) TGE group (TGE, 0.476 mg/g), (5) butyrate group (BA, 50 mM/200 μL), (6) treated group (TB) (TGE, 0.476 mg/g, and butyrate, 50 mM/200 μL) DAI evaluation and histological analysis IHC analysis: MPO activity in the colonic mucosa of mice (the rat anti-mouse MPO polyclonal antibody) Biomarkers of inflammatory condition: IL-6, IL-10, and TNF-α in the colon tissue (mouse-specific ELISA kits) Treg quantity: flow cytometry assay Analysis of butyrate in feces: GC-FID	Rectal administration of Turkish galls (TGE): - ↓ DAI scores vs. DSS control group (*p* < 0.001 for TGE and TB groups) - ↓ infiltration of inflammatory cells (*p* < 0.001 for TGE and TB groups vs. DSS control group; *p* < 0.05 for TB vs. TGE) - ↓ histopathological scores and inflammatory factors IL-6 and TNF-α (*p* < 0.001 and *p* < 0.01, respectively, for TGE and TB groups vs. DSS control group) - ↑ expression of IL-10 (*p* < 0.001 for TGE and TB groups vs. DSS control group) Turkish galls—alleviating UC by modulating gut microbiota: - ↓ harmful bacteria (Helicobacter, Bilophila, Acinetobacter, and Odoribacter) - ↑ putative SCFA-producing bacteria (Allobaculum, Bacteroides, Blautia, Butyricimonas) and butyrate concentration - ↑ anti-inflammatory bacteria (Bifidobacterium, Lactococcus)
[16]	China, 2019	Phytochemistry In vitro In vivo	The therapeutic effects of Turkish gall gallotannins (TGTs)-Fe^III^ microcapsules on DSS-induced UC in mice	TGTs—extracted using ethyl acetate TGTs-Fe^III^ microcapsules—prepared using TGT extract and FeCl_3_·6H_2_O	Identification of TGTs: LC-MS Characterizations of the TGTs-Fe^III^ microcapsules: UV, SEM, TEM, AFM, FT−IR, CLSM, and zeta potential measurements and with disassembly experiments UC mice model (Kunming mice, 4% DSS (*w*/*v*) in drinking water, for 7 days) Therapeutic effects on UC in mice: DAI evaluation of colitis; histopathological analysis of the colon tissue; biomarkers of inflammatory condition—IL-1β and TNF-α in the serum of mice (Elisa kit) Target effects on UC—adhesion experiments in vivo and ex vivo: IVIS fluorescence imager	A total of 9 constituents identified in TGTs’ ethyl acetate extract: 2 phenolic acids and 7 gallotannins Anti-inflammatory activity of TGTs-Fe^III^ microcapsules (enema suspension, 17 mg/kg/day) in DSS-induced experimental murine UC: - ↓ DAI scores vs. DSS group - ↓ histopathological scores vs. DSS group - ↓ proinflammatory cytokine levels: TNF-α and IL-1β - TGTs-Fe^III^ attached to the surface of the inflamed colon in both in vivo and ex vivo studies
[49]	Iraq, 2018	Phytochemistry In vitro	Phytochemical screening of the ethanolic and aqueous QIGE. Antihemolytic and antimicrobial activities of AgNPs of the extracts	QI galls—extracted with water or ethanol for 7 h AgNPs—synthesized using QIGE and an aqueous solution of silver nitrate in a 1:9 (*v*/*v*) ratio	Qualitative analysis: phytochemical tests Characterization of AgNPs: UV–Vis spectroscopy and SEM Antimicrobial activity against *E. coli*, *P. aeruginosa*, *S. aureus*, and *C. albicans*: agar well-diffusion method MIC: macrobroth dilution method Antihemolytic activity: free radical-induced erythrocyte lyses in rat blood	Phytochemical analysis: saponins, flavonoids, tannins, resins, alkaloids, glycosides, phenols, and coumarins AgNPs—sizes between 10 and 80 nm Antimicrobial activity: - AgNPs > QIGE - ethanolic and aqueous QIGE galls and their AgNPs—active against *E. coli*, *P*. *aeruginosa*, *S. aureus*, and *C. albicans* - the strongest effect on *P. aeruginosa*: inhibition zone reaching 30 and 25 mm, respectively, for extracts, and 35 and 30 mm, respectively, for AgNPs - MIC values of the extracts against each microorganism species: in the range of 0.2–0.4 g/mL QI galls’ extracts—weak antihemolytic activity
[50]	Iraq, 2018	Phytochemistry In vitro	Chemical constituents and anticancer effects of QIGE	QI galls—extracted with 70% ethanol in a ratio of 1:5 at 40 °C for 3 h	Identification of elements in QIGE: AAS Identification of bioactive compounds: GC-MS Cytotoxicity on mouse mammary carcinoma cell line 2003 (AMN3) and recombinant mouse epithelial cell line (L20B)	Chemical analysis: - A total of 9 elements and 34 compounds identified - the 3 main compounds: 2-hexanol, 2-methyl, 2,4-decadienal, and eucalyptol Cytotoxicity of QIGE: - ↓ AMN3 cancer cell line, IC_50_ of 0.2 mg/mL - ↓ L20B cell line with a maximum effect (45%) at 2 mg/mL - overgrowth at 200 mg/mL on L20B cancer cell line (no IC_50_)
[51]	China, 2018	Phytochemistry	Chemical constituents and antioxidant properties of the Turkish galls	Turkish gall powder (TGP)—with different particle sizes (>450, 400–250, 250–100, 100–50, and <50 μm): extracted with 50% methanol for 1 h at 1:50 (*w*/*v*) solid–liquid ratio	Phytochemical analysis: HPLC-DAD AA assays: DPPH; hydroxyl radical scavenging activity; superoxide radical scavenging activity Characterization of TGP: FT-IR; SEM; microscopic identification	Chemical analysis: - Three constituents identified and quantified: gallic acid, methyl gallate, and ellagic acid - ↑ content of gallic acid, methyl gallate, and ellagic acid in the extracts with ↓ particle size AA: - highly correlated with the contents of gallic acid, methyl gallate, and ellagic acid in the TGP extracts - the best activity corelated with particle size < 50 μm, while the lowest activity corelated with particle size > 450 μm
[52]	Malaysia, 2018	In vitro	Antimicrobial effects of the aqueous QIGE against pathogenic *Leptospira*	QI galls—extraction in water, in a ratio of 1:5 (*w*/*v*), at 50 °C for 72 h	MIC: microdilution broth assay MBC against the *L. interrogans* serovars Cell morphology of the extract-treated *L. interrogans* serovar Icterohaemorrhagiae: SEM	Antimicrobial inhibition of QIGE: - similar MIC values against both *L. interrogans* serovar Javanica and serovar Icterohaemorrhagiae (0.125 mg/mL) - MBC for *L. interrogans* serovar Javanica: 0.125 mg/mL - MBC for *L. interrogans* serovar Icterohaemorrhagiae: 0.250 mg/mL - SEM micrograph: showing changes in shape and size of the extract-treated cells (at 8 × MIC) vs. untreated cells
[21]	Egypt, 2018	Phytochemistry In vitro	Identification and quantification of phytochemical constituents in QIGE Antimicrobial action against *S. aureus*, *E. coli*, *P. aeruginosa*, *S. Typhimurium*, and *C. albicans* used for eggshell decontamination	QI (Olivier) galls—extraction in 70% ethanol in a ratio of 1:5 (*w*/*v*) for 6 h	Phytochemical analysis: HPLC-DAD Antimicrobial activity against *S. aureus* ATCC 25923, *E. coli* ATCC 25922, *P. aeruginosa* ATCC 25006, *S. Typhimurium* ATCC 23852, and *C. albicans* ATCC 1023: qualitative (ZOI) and quantitative (MIC) assays Morphology and viability of *S. aureus* cells: SEM	Chemical analysis: - A total of 22 phenolic compounds and 7 flavonoid compounds identified and quantified - the main phenolic compounds: p-hydroxybenzoic acid, pyrogallol, and catechol - the main flavonoid compounds: naringin and rutin Application of QIGE for disinfection of eggshells: - strong antimicrobial activity against contaminating microbial groups - the most sensitive strain—*S. aureus*, the most resistant—*S. Typhimurium* - complete inhibition of both *E. coli* and *S. aureus* after 60 min of immersion in 1% QIGE solution - sharply reduced total colony count, yeasts and molds, and Enterobacteriaceae (1.2, 2.5, and 0.3% remaining viable cells, respectively, after 60 min of immersion) - strong alterations in cell morphology of *S. aureus* cells treated with QIGE (the cells being entirely lysed and ruptured after 6 h of treatment)
[53]	Egypt, 2018	In vitro	Antimicrobial action of QIGE against skin pathogens, *S. aureus*, and *C. albicans*. Antimicrobial action durability of the QIGE treated textiles	QI galls—extraction in 70% ethanol in a ratio of 1:5 (*w*/*v*) for 350 min	Antimicrobial activity of QIGE compared to chitosan against different skin microbial pathogen strains: *C. albicans*-S (ATCC-10231), *C. albicans*-R (resistant strain to fluconazole, isolated from human skin lesion), *S. aureus*-S (ATCC-25923), and *S. aureus*-R (methicillin-resistant strain, isolated from infected wound)— agar well-diffusion method Durability of antimicrobial textiles (treated with 1% QIGE solution): repetitive laundering treatment	Antimicrobial activity of QIGE—evidenced against all skin pathogens examined: - relevant ZOIs of 10.6, 10.2, 11.7, and 11.2 mm against *C. albicans* S, *C. albicans* R, *S. aureus* S, and *S. aureus* R, respectively - fabrics treated with QIGE—higher antimicrobial action, against all strains, than chitosan treatments - *S. aureus* strains—more sensitive than *C. albicans* strains - antibiotic-sensitive isolates—more susceptible than antibiotic-resistant strains, toward the treated fabrics - the loaded textiles—maintaining 91.2 and 90.1% of their activity, after the 1st cycle, and 89.8 and 88.4% of the activity, respectively, after the 2nd cycle, compared to antibiotic-resistant microbial strains, *C. albicans* R and *S. aureus* R - the antimicrobial durability of loaded fabrics, against sensitive microbial strains—higher than that against resistant strains, for each microbial species and laundering cycle
[17]	China, 2018	Phytochemistry In silico In vitro In vivo	Chemical active constituents and intestinal anti-inflammatory effects of Turkish galls	TGP—reflux extraction with water, then with diethyl ether and ethyl acetate (Fr-A, Fr-B, Fr-C fractions)	Phytochemical analysis of ethyl acetate extracts (GEA): LC-MS In silico studies: system pharmacology approach In vitro studies: the screening of active constituents on RAW 264.7 mouse macrophage cells; cytotoxicity (MTT assay); NO production in LPS-stimulated RAW 264.7 cells; the expression of TNF-α, IL-1β, and IL-6 in the supernatant of RAW 264.7 cell (Elisa kit) In vivo study—DSS-induced experimental UC (Kunming mice, 4% DSS in drinking water, for 16 days): DAI evaluation and histopathological analysis; MPO assay in the colon tissue; expression of TNF-α, IL-1β, and IL-6 in the serum of mice (Elisa kit) Analysis of gene expression in colon and cell samples: qRT-PCR	Chemical analysis: - Nine constituents identified: phenolic acids and gallotannins In silico: 5 constituents (digallic acid, tri-*O*-galloyl-β-d-glucose, tetra-*O*-galloyl- β-d-glucose, penta-*O*-galloyl-β-d-glucose, and hexa-*O*-galloyl-β-d-glucose) hitting more than 5 potential targets and regulating multiple pathways, while all 9 constituents of GEA were involved in NF-κB pathway In vitro studies: - ↓ NO, IL-6, and TNF-α by the 5 major active constituents (methyl gallate, digallic acid, mono-*O*-galloyl-β-d-glucose, di-*O*-galloyl- β-d-glucose, and tri-*O*-galloyl-β-d-glucose) In vivo study: - Fr-A, Fr-B, Fr-C, and GEA (doses of 192 mg/kg for all): significant reduction in DAI score (*p* < 0.001) and body weight loss (*p* < 0.001), and bloody diarrhea symptoms, splenomegaly (*p* < 0.001), and histopathological scores of the mice (*p* < 0.001), vs. DSS group - Fr-B: significant reduction in MPO activity (*p* < 0.01) - Fr-A, Fr-B, and Fr-C: significant reduction in TNF-α (*p* < 0.001) and IL-1β (*p* < 0.001); Fr-B having the greatest efficacy - Fr-B: significant decrease in the expression of NF-κB-related genes (*p* < 0.05) in the colon (IL-1β, IL-6, TNF-α, ICAM-1, and TLR4) and in LPS-stimulated RAW 264.7 macrophage cells (IL-1β, IL-6, and ICAM-1); inhibition of NF-κB p65 protein expression in the colon and suppression of NF-κB p65 translocation to the nucleus

AA—antioxidant activity; AAS—atomic absorption spectrometry; ABTS—2,2′-azino-bis-3-ethylbenzothiazoline-6-sulfonic acid; AE—ascorbic acid equivalents; ALT—alanine transaminase; AOG—Aleppo oak gall; AST—aspartate transaminase; [Bmim][BF4]—1-Butyl-3-methylimidazolium tetrafluoroborate; [Bmim][Tf2N]—1-Butyl-3-methylimidazolium bis (trifluoromethylsulfonyl)imide; bw—body weight; CAE—conventional aqueous extraction; CAT—catalase; CE—catechine equivalents; CLSM—confocal laser confocal microscopy; CRC—colorectal cancer; CSE—conventional solvent extraction; CTAB—Hexadecyltrimethylammonium bromide; CUBAE—classical ultrasonic-bath assisted extraction; CUPRAC—Cupric Reducing Antioxidant Capacity; DAD—diode-array detector; DAI—disease activity index; DLS—dynamic light scattering; DMBA—7,12-dimethylbenz(a)anthracene; DPPH—2,2-diphenyl-1-picrylhydrazyl; DSS—dextran sulfate sodium; EDAX—energy dispersive X-ray; ESI—electrospray ionization; FESEM—field emission scanning electron microscopy; FID—flame ionization detector; FRAP—ferric reducing antioxidant power; FT-IR—Fourier transform infrared; GAE—gallic acid equivalents; GBM—glioblastoma multiforme; GC—gas chromatograph; GEA—ethyl acetate extracts of Turkish galls; HPLC—high-performance liquid chromatography; IC_50_—half-maximal inhibitory concentration; IHC—immunohistochemical; IL—interleukin; iNOS—inducible nitric oxide synthase; IR—infrared; IVIS—in vivo imaging system; LC_50_—50% lethality concentration; LC—liquid chromatography; LDH—lactate dehydrogenase; LH—luteinizing hormone; MBC—minimum bactericidal concentration; MCF-7—human breast adenocarcinoma; MCH—mean corpuscular hemoglobin; MCHC—mean corpuscular hemoglobin concentration; MDA—malondialdehyde; MIC—minimum inhibitory concentration; MPO—myeloperoxidase; MRSA—methicillin-resistant *Staphylococcus aureus;* MS—mass spectrometry; MTT—3-(4,5-dimethylthiazol-2-yl) -2,5-diphenyltetrazolium bromide; NF-κB—Nuclear factor-κB; NO—nitric oxide; NPs—nanoparticles; OGE—oak galls’ extract; OS—oxidative stress; PCL—polycaprolactone; PCR—quantitative real-time polymerase chain reaction; PDA—photodiode-array detector; PIDG—percentage inhibition of diameter growth; PVA—polyvinyl alcohol; QE—quercetin equivalents; QI—*Quercus infectoria*; QIEA—*Quercus infectoria* ethyl acetate extract; QIGE—*Quercus infectoria* gall extract; QILE—*Quercus infectoria* loaded emulsion; qRT-RT-PCR—reverse transcription polymerase chain reaction; ROS—reactive oxygen species; SCFAs—short-chain fatty acids; SCFE-CO_2_—supercritical fluid extraction CO_2_; SEM—scanning electron microscope; T—testosterone; T3—triiodothyronine; T4—thyroxin; TAC—total antioxidant capacity; TC—total cholesterol; TCT—total tannin content; TEM—transmission electron microscope; TE—trolox equivalents; TFC—total flavonoid content; TG—triglycerides; TGA—thermogravimetric analysis; TGF-β1— transforming growth factor beta 1; TGP—Turkish galls’ powder; TGTs—Turkish galls’ gallotannins; TLC—thin-layer chromatography; TLR4—Toll-like receptor 4; TNF-α—tumor necrosis factor-alpha; TPC—total phenolic content; Treg—regulatory T cells; TSH—thyroid-stimulating hormone; TTF-1—thyroid transcription factor-1; TTM—total thiol molecules; UC—ulcerative colitis; UPAE—ultrasonic-probe assisted extraction; UV—ultraviolet; Vis—visible; VEGF—vascular endothelial growth factor; XRD—X-ray diffraction; ZOI—zone of growth inhibition; ↓—decreased, ↑—increased.

**Table 2:** Phenolic compounds identified and quantified in galls of *Quercus* species.

Ref.	Extract	Analytical Method	Compounds	Amount
[10]	Methanolic extract of *Andricus sternlichti* galls	HPLC-DAD	Phenolic acids:	
			Gallic acid	7181.536 μg/g dw
			Ellagic acid	261,997.718 μg/g dw
			Caffeic acid	589,041.723 μg/g dw
			Chlorogenic acid	2375.615 μg/g dw
			*p*-Coumaric acid	635.284 μg/g dw
			Ferulic acid	1070.68 μg/g dw
			Cinnamic acid	747.044 μg/g dw
			Vanillic acid	16,466.952 μg/g dw
			3,4-Dihydroxybenzoic acid (syn. protocatechuic acid)	234.502 μg/g dw
			4-Hydroxybenzoic acid (syn. *p*-hydroxybenzoic acid	1223.13 μg/g dw
			2,5-Dihydroxybenzoic acid (syn. gentisic acid)	18,147.653 μg/g dw
			Flavonoids:	
			Epicatechin	171,497.57 μg/g dw
			Rutin	8156.209 μg/g dw
			Naringin	19,097.058 μg/g dw
			Quercetin	1.929 μg/g dw
[9]	Methanolic extract of QI galls obtained using CSE	LC-MS/MS	Phenolic acids:	NA
			Gallic acid	
			Ellagic acid	
			Quinic acid	
			Hydrolysable tannins–gallotannins:	NA
			Tannic acid (syn. gallotannin)	
	Extract obtained using SCFE-CO_2_		Phenolic acids:	NA
			Gallic acid	
			Ellagic acid	
			Salicylic acid	NQ
			Chlorogenic acid	
			Caffeic acid	
			Flavonoids:	NQ
			Myricetin	
			Quercetin	
			Apigenin	
			Hydrolysable tannins–gallotannins:	NA
			Tannic acid	
	Extract obtained using SCFE-CO_2_ with methanol co-solvent		Phenolic acids:	NA
			Gallic acid	
			Ellagic acid	
			Quinic acid	
			Salicylic acid	NQ
			Chlorogenic acid	
			*Trans*-caffeic acid	
			*p*-Coumaric acid	
			Rosmarinic acid	
			Flavonoids:	NQ
			Myricetin	
			Quercetin	
			Apigenin	
			Rutin	
			Hesperidin	
			Hyperoside	
			Fisetin	
			Naringenin	
			Hesperetin	
			Luteolin	
			Kaempferol	
			Rhamnetin	
			Chrysin	
			Benzaldehydes:	NQ
			Vanillin	
			Coumarins:	NQ
			Coumarin	
			Hydrolysable tannins–gallotannins:	NA
			Tannic acid	
[8]	50% ethanolic extract of QI (Olivier) galls	HPLC-DAD	Hydrolysable tannins–gallotannins:	
			Tannic acid	403 mg/g dw
			Phenolic acids:	
			Gallic acid	291 mg/g dw
			Ellagic acid	131 mg/g dw
[14]	Ethanolic extract of QI galls	LC-MS/MS	Hydrolysable tannins–gallotannins:	NQ
			Monogalloyl glucose	
			Galloyl glyceride	
			Digalloyl glucose I	
			Digalloyl glucose II	
			Trigalloyl glucose I	
			Trigalloyl glucose II	
			Tetra galloyl glucose	
			Penta galloyl glucose	
			Methyl gallate	
			Phenolic acids and their methyl esters:	NQ
			Gallic acid	
			Dihydroxy benzoic acid	
			Ellagic acid	
			Quinic acid	
			2-*O*-Galloyl hydroxymalonic acid	
			Syringic acid	
			*m*-Digallic acid	
			*p*-Digallic acid	
			Digallic methyl ester	
			Digallic dimethyl ester	
			Trigallic dimethyl ester	
[15]	Aqueous extract obtained using SE (for identification). Methanol-enriched fractions F1-F6 of QI aqueous crude extract (for quantification)	TLC; HPLC-DAD	Hydrolysable tannins–gallotannins:	
			Gallotannin (F1)	56.1 ± 12.2 mg/g dw ^a^
			Gallotannin (F2)	70.8 ± 8.8 mg/g dw
			Gallotannin (F3)	374.4 ± 29.8 mg/g dw
			Gallotannin (F4)	711.5 ± 32.1 mg/g dw
			Gallotannin (F5)	470.6 ± 16.4 mg/g dw
			Gallotannin (F6)	39.5 ± 10.5 mg/g dw
[33]		TLC; HPLC-PDA	Hydrolysable tannins–gallotannins:	
	Aqueous extract of QI galls obtained using SE		Gallotannin	75.0 mg/g dw
	Methanolic extract of QI galls obtained using SE		Gallotannin	46.8 mg/g dw
[34]	70% ethanolic extract of QI galls	HPLC-PDA	Phenolic acids:	
			Gallic acid	12.30 ± 0.9 mg/g dw ^a^
			Caffeic acid	3.94 ± 0.2 mg/g dw
			Flavonoids:	
			Rutin	10.72 ± 0.7 mg/g dw
			Quercetin	5.00 ± 0.3 mg/g dw
[40]	Methanolic extract of *A. tomentosus* galls	HPLC-DAD	Phenolic acids:	
			Gallic acid	7218.09 μg/g dw
			Ellagic acid	187,696.132 μg/g dw
			Caffeic acid	424,068.479 μg/g dw
			Chlorogenic acid	1013.789 μg/g dw
			*p*-Coumaric acid	151.081 μg/g dw
			Ferulic acid	100.731 μg/g dw
			Cinnamic acid	423.675 μg/g dw
			Vanillic acid	6572.271 μg/g dw
			3,4-Dihydroxybenzoic acid	3109.659 μg/g dw
			4-Hydroxybenzoic acid	3859.173 μg/g dw
			2,5-Dihydroxybenzoic acid	69,399.147 μg/g dw
			Flavonoids:	
			Epicatechin	53,430.17 μg/g dw
			Rutin	337.586 μg/g dw
			Naringin	315.325 μg/g dw
			Quercetin	1141.256 μg/g dw
[18]	Aqueous extract of Turkish galls	HPLC-ESI-MS/MS	Phenolic acids:	NQ
			Gallic acid	
			Digallic acid 1	
			Digallic acid 2	
			Ellagic acid	
			Hydrolysable tannins–gallotannins:	NQ
			Monogalloyl-glucoside	
			Digalloyl-glucoside	
			Trigalloyl-glucoside 1	
			Trigalloyl-glucoside 2	
			Trigalloyl-glucoside 3	
			Trigalloyl-glucoside 4	
			Tetragalloyl-glucoside 1	
			Tetragalloyl-glucoside 2	
			Pentagalloyl-glucoside 1	
			Pentagalloyl-glucoside 2	
			Hexagalloyl-glucoside 1	
			Hexagalloyl-glucoside 2	
			Heptagalloyl-glucoside	
			Hydrolysable tannins–ellagitannins:	NQ
			Galloyl-HHDP-glucose	
			Pedunculagin	
[19]	Methanolic extract of QI galls	GC-MS	Flavonoids:	NQ
			Lucenin 2	
			Hydrolysable tannins–gallotannins:	NQ
			Benzoic acid, 3,4,5-trihydroxy, methyl ester (syn. methyl gallate)	
			Hydroxyphenol derivatives:	NQ
			2-Allyl-5-t-butylhydroquinone	
			Dihydroxyphenols and derivatives:	NQ
			1,2,3-Benzenetriol (syn. pyrogallol)	
			1,2-Benzenediol, 3-methoxy (syn. pyrocatechol)	
[43]	Aqueous extract of oak galls obtained using CAE, with and without the presence of ionic liquid	HPLC-DAD	Phenolic acids:	
			Gallic acid	25.34–43.76 mg/g dw
			Ellagic acid	7.33–14.15 mg/g dw
			Salicylic acid	0.61–1.14 mg/g dw
			Chlorogenic acid	4.04–8.57 mg/g dw
			Caffeic acid	0.50–2.01 mg/g dw
			*Flavonoids:*	
			Myricetin	0.05–0.06 mg/g dw
			Quercetin	0.03–0.16 mg/g dw
			Apigenin	NQ
			Hydrolysable tannins–gallotannins:	
			Tannic acid	98.86–179.97 mg/g dw
	Aqueous extract of oak galls obtained using CUBAE, with and without the presence of ionic liquid		Phenolic acids:	
			Gallic acid	42.35–81.56 mg/g dw
			Ellagic acid	14.25–19.56 mg/g dw
			Salicylic acid	1.05–6.09 mg/g dw
			Chlorogenic acid	5.67–10.43 mg/g dw
			Caffeic acid	1.40–5.53 mg/g dw
			Flavonoids:	
			Myricetin	0.05–0.12 mg/g dw
			Quercetin	0.08–0.76 mg/g dw
			Apigenin	0.01–0.03 mg/g dw
			Hydrolysable tannins–gallotannins:	
			Tannic acid	228.76–810.74 mg/g dw
	Extracts of oak galls in water, methanol, CTAB, and 2 ionic liquids, [Bmim][BF4] and [Bmim][Tf2N], obtained using UPAE		Phenolic acids:	
			Gallic acid	65.04–130.76 mg/g dw
			Ellagic acid	16.11–33.44 mg/g dw
			Salicylic acid	3.69–6.61 mg/g dw
			Chlorogenic acid	8.13–17.23 mg/g dw
			Caffeic acid	3.56–10.07 mg/g dw
			Flavonoids:	
			Myricetin	0.09–0.55 mg/g dw
			Quercetin	0.47–3.76 mg/g dw
			Apigenin	0.03–0.09 mg/g dw
			Hydrolysable tannins–gallotannins:	
			Tannic acid	776.75–1556.26 mg/g dw
[20]	Extracts of QI galls in ethyl acetate, n-butanol, ethanol, and water	LC-MS/MS	Phenolic acids:	NA
			Protocatechuic acid	
			Chlorogenic acid	
			Flavonoids:	
			Luteolin-7-glucoside	
			Rutin	
			Hesperidin	
			Hyperoside	
			Apigetrin	
			Quercitrin	
			Astragalin	
			Quercetin	
			Luteolin	
			Apigenin	
			Naphthodianthrones:	
			Pseudohypericin	
			Hypericin	
			Prenylated phloroglucinol derivatives:	
			Hyperforin	
[47]		HPLC-DAD	Hydrolysable tannins–gallotannins:	
	96% ethanolic extract of QI subsp. *infectoria* galls		Tannic acid	59.033–81.012 mg/g dw
	80% ethanolic extract of QI subsp. *infectoria* galls		Tannic acid	43.898–127.683 mg/g dw
	70% acetone extract of QI subsp. *infectoria* galls		Tannic acid	3.064–67.200 mg/g dw
	Extracts of QI subsp. *infectoria* galls in diethylether/ethanol/water (25:3:1)		Tannic acid	0.04–0.112 mg/g dw
	96% ethanolic extract of QI subsp. *boissieri* galls		Tannic acid	30.852 mg/g dw
	80% ethanolic extract of QI subsp. *boissieri* galls		Tannic acid	52.846 mg/g dw
	70% acetone extract of QI subsp. *boissieri* galls		Tannic acid	37.602 mg/g dw
	Extracts of QI subsp. *boissieri* galls in diethylether/ethanol/water (25:3:1)		Tannic acid	0.016 mg/g dw
[16]	Ethyl acetate extract of QI (Olivier) galls	LC-MS	Phenolic acids:	NQ
			Gallic acid	
			Digallic acid	
			Hydrolysable tannins–gallotannins:	
			Mono-*O*-galloyl-β-d-glucose	
			Di-*O*-galloyl-β-d-glucose	
			Tri-*O*-galloyl-β-d-glucose	
			Tetra-*O*-galloyl-β-d-glucose	
			Penta-*O*-galloyl-β-d-glucose	
			Hexa-*O*-galloyl-β-d-glucose	
			Hepta-*O*-galloyl-β-d-glucose	
[51]	50% methanolic extract of Turkish galls with TGP particle size:	HPLC-DAD	Phenolic acids:	
	>450 μm		Gallic acid	7.82 ± 0.04 mg/g dw ^a^
	400–250 μm		Gallic acid	8.29 ± 0.05 mg/g dw
	250–100 μm		Gallic acid	8.41 ± 0.04 mg/g dw
	100–50 μm		Gallic acid	8.67 ± 0.11 mg/g dw
	<50 μm		Gallic acid	9.47 ± 0.09 mg/g dw
	>450 μm		Ellagic acid	0.64 ± 0.004 mg/g dw
	400–250 μm		Ellagic acid	0.65 ± 0.003 mg/g dw
	250–100 μm		Ellagic acid	0.69 ± 0.005 mg/g dw
	100–50 μm		Ellagic acid	0.75 ± 0.002 mg/g dw
	<50 μm		Ellagic acid	0.79 ± 0.004 mg/g dw
			Hydrolysable tannins–gallotannins:	
	>450 μm		Methyl gallate	26.07 ± 0.18 mg/g dw
	400–250 μm		Methyl gallate	28.68 ± 0.19 mg/g dw
	250–100 μm		Methyl gallate	30.23 ± 0.22 mg/g dw
	100–50 μm		Methyl gallate	33.87 ± 0.31 mg/g dw
	<50 μm		Methyl gallate	34.78 ± 0.35 mg/g dw
[21]	70% ethanolic extract of QI (Olivier) galls	HPLC-DAD	Phenolic acids:	
			*p*-Hydroxybenzoic	74,473.96 μg/g dw
			e-Vanillic acid	15,012.16 μg/g dw
			Vanillic acid	9518.89 μg/g dw
			Chlorogenic acid	8887.12 μg/g dw
			Caffeic acid	7667.85 μg/g dw
			Protocatechuic acid	3768.09 μg/g dw
			Isoferulic acid	1928.71 μg/g dw
			Ellagic acid	1146.86 μg/g dw
			Alpha-coumaric acid	803.21 μg/g dw
			Ferulic acid	751.61 μg/g dw
			Gallic acid	364.76 μg/g dw
			*p*-Coumaric acid	171.26 μg/g dw
			Cinnamic acid	49.18 μg/g dw
			Rosmarinic acid	16.33 μg/g dw
			Flavonoids:	
			Catechin	15,622.42 μg/g dw
			Naringin	123.22 μg/g dw
			Rutin	103.25 μg/g dw
			Quercitrin	89.82 μg/g dw
			Quercetin	14.27 μg/g dw
			Hesperetin	4.66 μg/g dw
			7-Hydroxyflavone	3.50 μg/g dw
			Dihydroxyphenols:	
			Pyrogallol	71,666.14 μg/g dw
			Hydroxyphenols:	
			Catechol	66,966.37 μg/g dw
			Phenolic alcohols:	
			3-Hydroxytyrosol	10,384.97 μg/g dw
			Coumarins:	
			Coumarin	557.30 μg/g dw
			Stilbenes:	
			Resveratrol	469.84 μg/g dw
[17]	Extract of Turkish galls in water/diethyl ether/ethyl acetate	LC-MS	Phenolic acids:	NQ
			Gallic acid	
			Digallic acid	
			Hydrolysable tannins–gallotannins:	
			Mono-*O*-galloyl-β-d-glucose	
			Di-*O*-galloyl-β-d-glucose	
			Tri-*O*-galloyl-β-d-glucose	
			Tetra-*O*-galloyl-β-d-glucose	
			Penta-*O*-galloyl-β-d-glucose	
			Hexa-*O*-galloyl-β-d-glucose	
			Methyl gallate	

[Bmim][BF4]—1-Butyl-3-methylimidazolium tetrafluoroborate; [Bmim][Tf2N]—1-Butyl-3-methylimidazolium bis (trifluoromethylsulfonyl)imide; CAE—conventional aqueous extraction; CSE— conventional solvent extraction; CTAB—Hexadecyltrimethylammonium bromide; CUBAE—classical ultrasonic-bath assisted extraction; F1—0% methanol fraction; F2—10% methanol fraction; F3—25% methanol fraction; F4—50% methanol fraction; F5—75% methanol fraction; F6—100% methanol fraction; HPLC-DAD—high-performance liquid chromatography with diode-array detection; HPLC-ESI-MS/MS—high-performance liquid chromatography–electrospray mass spectrometry; HPLC-PDA—high-performance liquid chromatography coupled with a photodiode-array detector; LC-MS—liquid chromatography with mass spectrometry; LC-MS/MS—liquid chromatography with tandem mass spectrometry; NA—not available (the amount of compound is not expressed in relation to the mass of dry oak galls); NQ—non-quantified; QI—*Quercus infectoria*; SCFE-CO_2_—supercritical fluid extraction CO_2_; SE—soxhlet extraction; TGP—Turkish galls’ powder; TLC—thin-layer chromatography; UPAE—ultrasonic-probe assisted extraction; ^a^—the amounts are presented as means ± SD (standard deviation); dw—dry weight.

**Table 3:** Other compounds identified and quantified in galls of *Quercus* species.

Classification	Compound	Amount	Analytical Method	Ref.
Hydrocarbons	Tetratetracontane	NQ	GC-MS	[19]
	Triacontane			
	Dotriacontane			
	Nonacosane			
Dicarboxylic acids	Malic acid	9.75–79.28 mg/g dw	HPLC-DAD	[43]
		NQ	LC-MS/MS	[9]
Dicarboxylic acid derivatives	2,3-Dimethyl fumaric acid	NQ	GC-MS	[19]
Tricarboxylic acids	Aconitic acid	4.31–20.37 mg/g dw	HPLC-DAD	[43]
		NQ	LC-MS/MS	[9]
Aromatic carboxylic acids	Benzoic acid	9.25 ± 0.6 mg/g dw ^a^	HPLC-PDA	[34]
		0.23–7.54 mg/g dw	HPLC-DAD	[43]
		1414.21 μg/g dw	HPLC-DAD	[21]
		NQ	LC-MS/MS	[9]
Fatty acids	Hexanoic acid	NQ	GC-MS	[50]
	Octanoic Acid			
	Hexadecanoic acid	NQ	GC-MS	[19]
	Octadecenoic acid			
	9-Octadecenoic acid			
Aromatic amino acids	4-Amino-benzoic acid	495.97 μg/g dw	HPLC-DAD	[21]
Fatty amides	13-Docosenamide	NQ	GC-MS	[19]
Fatty aldehydes	Hexanal (syn. Caproaldehyde)	NQ	GC-MS	[50]
	2-Heptenal, (Z)-			
	2-Octenal, (E)-			
	2-Nonenal, (E)-			
	2-Decenal, (Z)-			
	2,4-Decadienal, (E,E)-			
	2,4-Decadienal			
Cinnamaldehydes	2-Propenal, 3-phenyl-	NQ	GC-MS	[50]
Aliphatic alcohols	2-Hexanol, 2-methyl-	NQ	GC-MS	[50]
	Ethanol, pentamethyl-			
	Hexahydrofarnesol	NQ	GC-MS	[19]
Monoterpenes	(+)-m-Mentha-1(6),8-diene (syn. Sylvestrene)	NQ	GC-MS	[50]
	1,4-Cyclohexadiene, 1-methyl-4-(1-methylethyl)- (syn. γ-Terpinene)			
	(+)-4-Carene			
	1,3,8-p-Menthatriene			
Sesquiterpenes	Copaene	NQ	GC-MS	[50]
	1H-Cycloprop(e)azulene, 1a,2,3,4,4a,5,6,7b-octahydro-1,1,4,7-tetramethyl-, (1aR,4R,4aR,7bS)-			
	4,8,8-Trimethyl-2-methylene-4-vinylbicyclo[5.2.0]nonane			
	1H-Cycloprop[e]azulene, decahydro-1,1,7-trimethyl-4-methylene (syn. Aromadendrene)			
	1,4-Methanoindan, hexahydro-7-isopropyl-4-methyl-8-methylene- (syn. (+)-Sativen)			
	Humulane-1,6-dien-3-ol			
Monoterpenoids	Eucalyptol	NQ	GC-MS	[50]
	2(10)-Pinen-3-one, (+/−)- (syn. Pinocarvone)			
	p-Menth-1-en-4-ol			
	2-Isopropenyl-5-methylhex-4-enal			
	p-Menth-1-en-8-ol, (S)-(-)-(syn. (S)-(-)-α-Terpineol)			
	Eugenol			
Diterpenoids	Kaur-16-ene	NQ	GC-MS	[50]
Ethers	Anisole, p-propenyl- (syn. *trans*-anethole)	NQ	GC-MS	[50]
	2,2,3,3-Tetraethyloxirane			
Esters	Pyrrole-2-carboxylic acid, 4-(1-chlorodec-1-enyl)-3,5-dimethyl-, ethyl ester	NQ	GC-MS	[50]
	Methyl 11-octadecenoate			
Thiocyanates	Adamantane 1-thiocyanatomethyl-	NQ	GC-MS	[50]
Triazoles	4H-1,2,4-Triazol-3-amine, 4-methyl	NQ	GC-MS	[19]
Methylxanthines	Caffeine	21,676.51 μg/g dw	HPLC-DAD	[21]
Elements	Fe	NA	AAS	[50]
	Zn			
	Cu			
	Mn			
	K			
	Cd			
	Co			
	Ti			
	N	NA	Macro Kjeldahl method	[50]
Proteins		NA	Macro Kjeldahl method	[50]

AAS—atomic absorption spectrometry; GC-MS—gas chromatography–mass spectrometry; HPLC-DAD—high-performance liquid chromatography with diode-array detection; NA—not available (the amount of compound is not expressed in relation to the mass of dry oak galls); NQ—non-quantified; ^a^—the amounts are presented as means ± SD (standard deviation); dw—dry weight.

## Data Availability

Data are contained within the article.
